# Breaking genetic shackles: The advance of base editing in genetic disorder treatment

**DOI:** 10.3389/fphar.2024.1364135

**Published:** 2024-03-06

**Authors:** Fang Xu, Caiyan Zheng, Weihui Xu, Shiyao Zhang, Shanshan Liu, Xiaopeng Chen, Kai Yao

**Affiliations:** ^1^ Institute of Visual Neuroscience and Stem Cell Engineering, Wuhan University of Science and Technology, Wuhan, China; ^2^ College of Life Sciences and Health, Wuhan University of Science and Technology, Wuhan, China

**Keywords:** base editing, cytosine base editors, adenine base editors, delivery strategies, genetic diseases

## Abstract

The rapid evolution of gene editing technology has markedly improved the outlook for treating genetic diseases. Base editing, recognized as an exceptionally precise genetic modification tool, is emerging as a focus in the realm of genetic disease therapy. We provide a comprehensive overview of the fundamental principles and delivery methods of cytosine base editors (CBE), adenine base editors (ABE), and RNA base editors, with a particular focus on their applications and recent research advances in the treatment of genetic diseases. We have also explored the potential challenges faced by base editing technology in treatment, including aspects such as targeting specificity, safety, and efficacy, and have enumerated a series of possible solutions to propel the clinical translation of base editing technology. In conclusion, this article not only underscores the present state of base editing technology but also envisions its tremendous potential in the future, providing a novel perspective on the treatment of genetic diseases. It underscores the vast potential of base editing technology in the realm of genetic medicine, providing support for the progression of gene medicine and the development of innovative approaches to genetic disease therapy.

## 1 Introduction

Over the past few decades, there have been remarkable strides in deciphering and exploring the human genome, leading to a profound comprehension of its intricacies. The advent of high-throughput sequencing technologies has greatly facilitated the mapping of a multitude of genes and their associated variants, numbering in the tens of thousands. Among the myriad types of genetic mutations, single nucleotide variations (SNVs) stand out as the most prevalent and widely distributed mutations across the genome (Abecasis et al., 2010). Although the majority of these variations do not manifest discernible effects or alter gene functionality, particular mutations lead to significant phenotypic changes and have been implicated in a broad spectrum of diseases (Collins et al., 1997). It is estimated that roughly half of all hereditary variations can be attributed to SNVs, which have the capacity to disrupt the functionality of protein-coding genes and contribute to the onset of diseases (Bamshad et al., 2011; Sun and Yu, 2019; Porto et al., 2020). Hence, the exploration of technologies with the ability to directly rectify or modify pathogenic genes harbors substantial potential in elucidating the mechanisms underlying human hereditary disorders. Within this context, the emergence of gene editing techniques has bestowed upon us a powerful arsenal for controlling and rectifying genomic variations, thus revolutionizing our capacity to address genetic anomalies.

**FIGURE 1:**
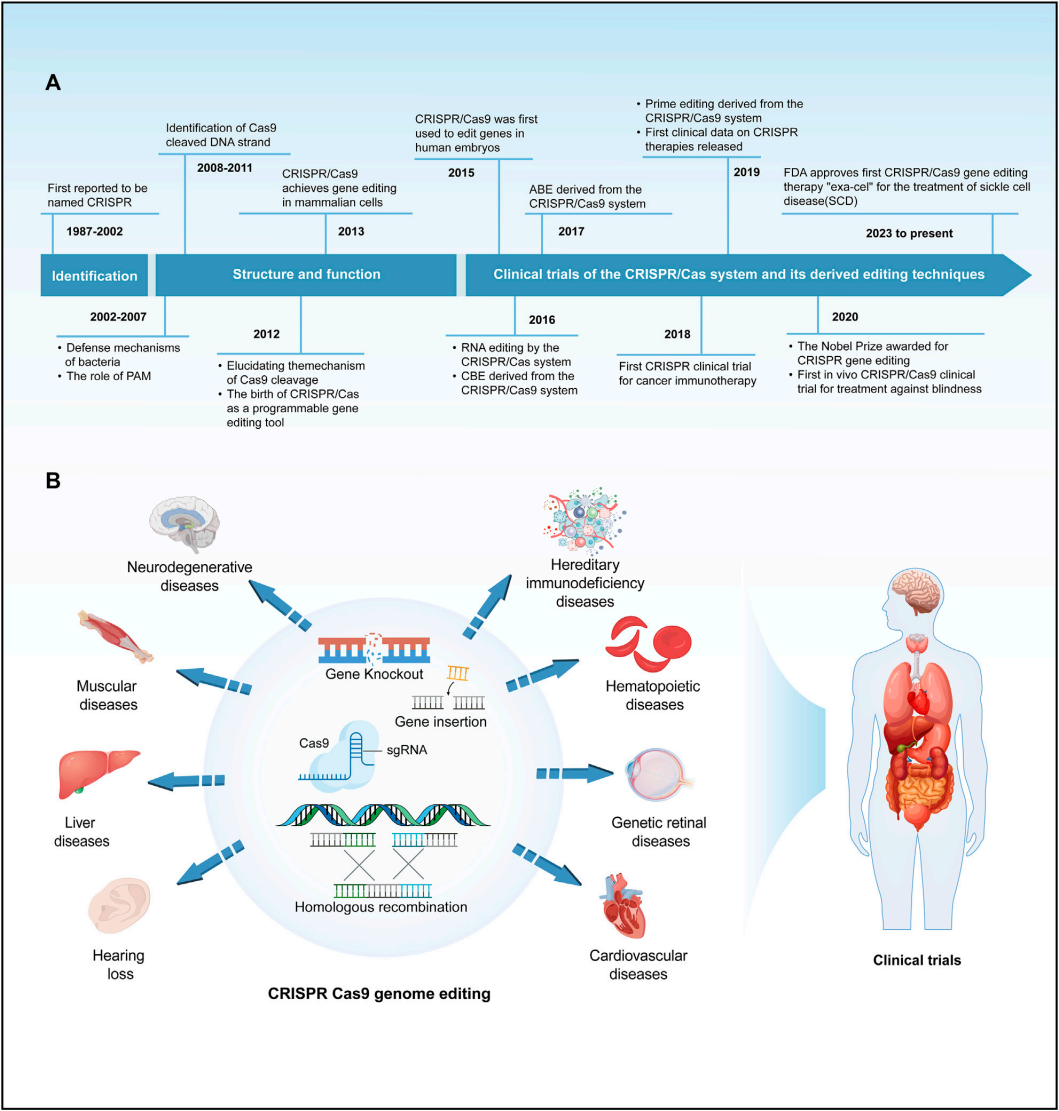
A brief history of the development and application of the CRISPR/Cas system. **(A)** Provides a concise overview of the timeline of key events in the development of the CRISPR/Cas system. **(B)** Summarize the main applications and clinical trials of the CRISPR/Cas9 system in the field of genetic diseases.

Gene editing is a revolutionary biotechnology that can be traced back to DNA recombinant technology in the 1970s. During that era, scientists initiated the use of restriction enzymes to cleave and reassemble DNA fragments, laying the groundwork for subsequent gene editing methodologies. Traditional genetic engineering methods relied on restriction enzymes for cleaving and fusing DNA fragments, yet these techniques were complex and had inherent limitations. In the early 21st century, scientists spearheaded the development of a repertoire of programmable nucleases for genome editing, encompassing meganucleases (Stoddard, 2011), zinc-finger nucleases (ZFN) (Urnov et al., 2010), transcription activator-like effector nucleases (TALENs) (Bogdanove and Voytas, 2011; Scharenberg et al., 2013) and leveraging the clustered regularly interspaced short palindromic repeats/CRISPR-associated protein 9 (CRISPR/Cas9) system for precise gene editing (Mali et al., 2013; Hsu et al., 2014). These advancements ushered in a new era of gene editing characterized by heightened precision and efficiency. Meganucleases, ZFNs, and TALENs necessitate protein engineering for the creation of specific sequence-binding domains, a process that is intricate and costly. In contrast, the CRISPR/Cas9 system employs RNA-guided gene editing, streamlining the design and construction of target sequences and eliminating the labor-intensive process of protein design (Cox et al., 2015). The CRISPR/Cas9 system consists of a single-guide RNA (sgRNA) and a Cas9 protein with endonuclease activity. Under the specific recognition of sgRNA, the Cas9 protein reaches the specific site in the genome, causing double-stranded DNA (dsDNA) breaks. Double-strand breaks are predominantly mended via two cellular pathways: the inherent nonhomologous end joining (NHEJ) and the homology-directed repair (HDR), the latter being instrumental in precise genomic alterations (Terns and Terns, 2011; Cong et al., 2013). Since the discovery of CRISPR sequences, CRISPR/Cas9 technology has significantly advanced research in the life sciences, offering unprecedented new approaches for treating human diseases (Haydar et al., 2020; Ledford, 2020; Stadtmauer et al., 2020; Frangoul et al., 2021; Sheridan, 2024) (Figure 1A). This revolutionary technology has been applied in the research and treatment of a wide array of diseases, including but not limited to blood disorders, liver diseases, hearing loss, and numerous rare genetic conditions (Long et al., 2016; Kolli et al., 2017; Bjursell et al., 2018; Charlesworth et al., 2018; Gu et al., 2022) (Figure 1B). The clinical application of CRISPR/Cas9 technology has shown promising results in treating these diseases, particularly achieving notable progress in areas such as genetic retinal diseases, hereditary immunodeficiency diseases, hereditary cardiovascular diseases, β-thalassemia, and sickle cell disease (SCD) (Haydar et al., 2020; Gillmore et al., 2021; Cowan et al., 2022; Russell et al., 2022; Sharma et al., 2023; Longhurst et al., 2024) (Figure 1B). Despite its revolutionary impact, CRISPR/Cas9 is hindered by the limited efficiency of HDR, primarily active in mitotic cells, often defaulting to NHEJ. This proclivity towards NHEJ raises the likelihood of nucleotide insertions and deletions (indels), presenting a significant challenge in the pursuit of precision in genome editing (Cong et al., 2013; Cong and Zhang, 2015). Furthermore, the CRISPR/Cas9 system has raised critical issues regarding potential immune responses induced by the introduction of foreign Cas9 proteins and sgRNA molecules, alongside concerns about the long-term safety of gene editing (Li and Li, 2020). These issues necessitate in-depth research to enhance editing precision, minimize immune responses to the greatest extent possible, and understand the enduring impacts of genomic alterations, thereby ensuring the safety and efficacy of CRISPR applications. Consequently, to precisely correct pathogenic gene point mutations, it is essential to enhance the efficiency of CRISPR/Cas9 systems and develop more reliable predictive mechanisms. Base editors represent a burgeoning gene editing tool capable of directly modifying specific bases within DNA or RNA sequences. This capacity for targeted alteration in an organism’s genome or transcriptome allows for refined regulation of gene functionality and expression (Porto et al., 2020). As research and development in this area continue to progress, base editors are poised to revolutionize the approach to genetic disease treatment. They offer hope for effective therapies where traditional treatments have been limited or non-existent. This technology not only has the potential to improve the quality of life for individuals with genetic disorders but also represents a significant stride forward in the broader pursuit of advancing human health.

The article presents a thorough compilation of the fundamental principles and delivery methods of DNA and RNA base editors, with a specific emphasis on their potential applications in treating hereditary diseases. This comprehensive review not only emphasizes the current state of base editing technology but also explores its future possibilities, highlighting its significance and potential impact in the field of genetic disease treatment, thereby offering a new perspective for the treatment of genetic disorders.

## 2 Base editing in DNA

**FIGURE 2:**
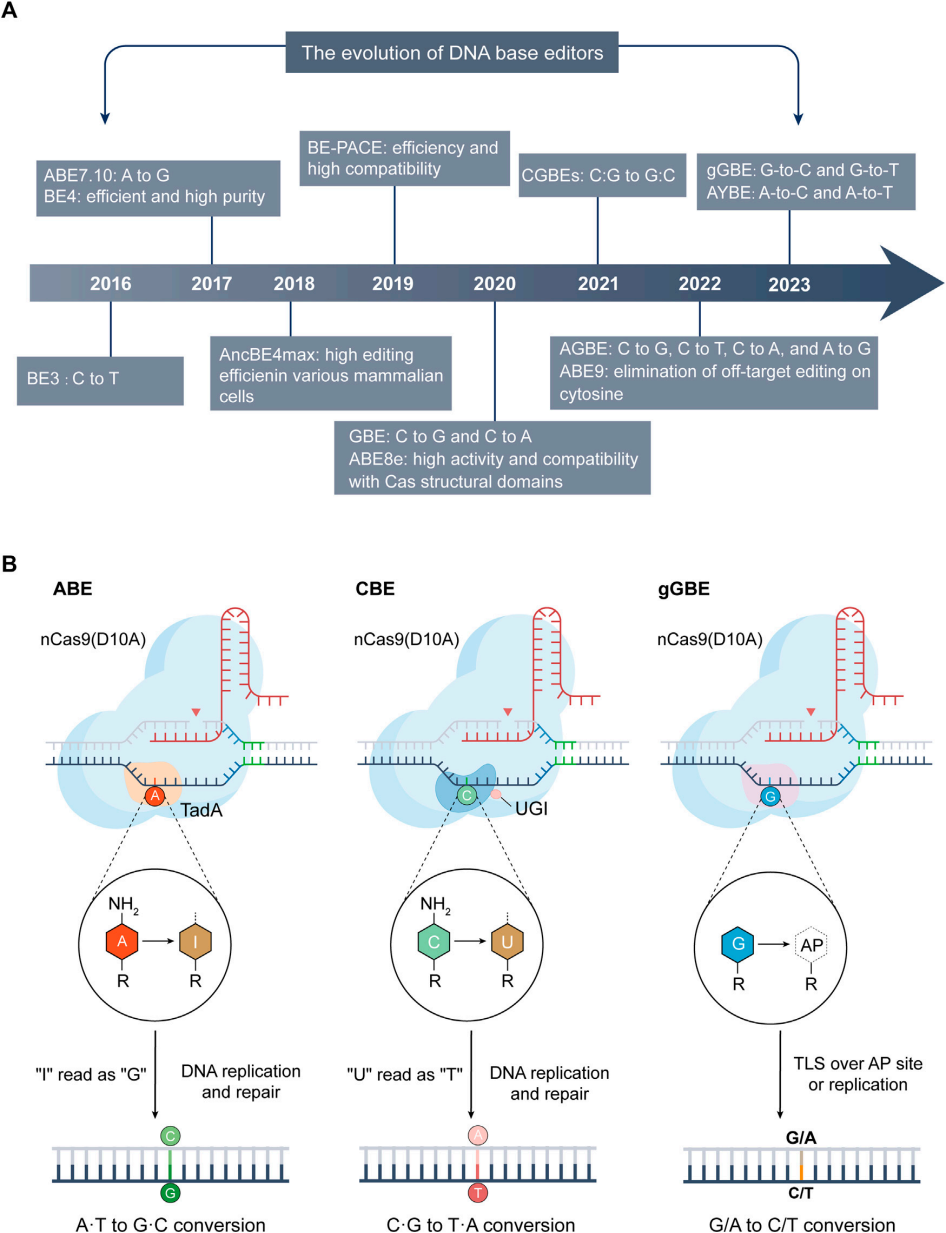
The advancements and mechanisms of base editing technologies. **(A)** Evolution of DNA base editing (2016–2023): This section maps the significant developments and innovations in base editing technology from 2016 to 2023. (B) Mechanisms of ABE (left), CBE (center), and the latest deaminase-free glycosylase-based guanine base editor (gGBE, right): A common feature of these editors is the inclusion of their respective Cas9 variants and sgRNA. ABE utilizes variants of adenine deaminase (like TadA), converting A into inosine (I), which is typically read as G during DNA repair or replication, thus achieving A-to-G conversion. CBE employs cytosine deaminases (such as APOBEC1) or its variants to deaminate C into U. During DNA repair or replication, U is usually read as T, enabling C-to-T conversion. The guanine base editor (gGBE) employs N-methylpurine DNA glycosylase (MPG) to recognize and remove G from the DNA strand, and the resulting apurinic/apyrimidinic (AP) site are subsequently repaired through translesion synthesis (TLS) or DNA replication, culminating in G-to-C or G-to-T transversions.

DNA base editors, comprising cytosine base editors (CBEs) (Komor et al., 2016) and adenine base editors (ABEs) (Gaudelli et al., 2017), are distinguished by their capacity for precise point mutations, circumventing the need for donor DNA templates or the induction of double-strand breaks. This technological advancement facilitates targeted modifications at the genomic base level (Figure 2A), ensuring the preservation of the surrounding genetic sequence’s integrity.

### 2.1 Cytosine base editors (CBEs)

The cytosine base editor (CBE), an evolution of the CRISPR/Cas9 system, known as the base editor (BE), consists of two essential components: a single-guide RNA (sgRNA) and a fusion protein comprising a Cas9 variant lacking double-strand cleavage activity (dCas9 or nCas9) and a cytosine deaminase enzyme (Komor et al., 2016). dCas9 (dead Cas9) and nCas9 (nickase Cas9) are generated through mutations in the RuvC and HNH domains of the Cas9 nuclease (Jinek et al., 2012). dCas9 preserves DNA binding without cleaving the backbone, while nCas9 exclusively cleaves one DNA strand, causing a single-strand break. By using Cas9 proteins that have lost cleavage activity or have only one strand of cleavage activity to achieve targeted base substitutions at the target site, the base editing system does not rely on double-strand breaks formation. The fusion protein, guided to genomic DNA by sgRNA, enables cytosine deaminase to transform cytosine (C) into uracil (U), which is then changed to thymine (T) during DNA repair or replication, effectuating a C to T base conversion (Figure 2B) (Komor et al., 2016). Cytidine deaminases in nature predominantly exhibit enzymatic activity on RNA. In the quest to identify a suitable DNA cytidine deaminase for base editing applications, researchers conducted evaluations on the single-stranded DNA (ssDNA) deamination activities of various naturally occurring cytidine deaminases (hAID, hAPOBEC3G, rAPOBEC1, and pmCDA1). Among these variants, rAPOBEC1, originating from rats, exhibited the highest deamination activity on ssDNA. The inaugural base editor, BE1 (rAPOBEC1–XTEN–dCas9), integrates rAPOBEC1 attached to the N-terminus of dCas9 via an XTEN linker, composed of 16 residues (Komor et al., 2016). *In vitro*, BE1 exhibits a base editing efficiency between 25% and 40%, with its activity covering roughly a 5-nucleotide (nt) window, initiating from the fourth to eighth position when counted from the most distal end of the protospacer adjacent motif (PAM) sequence. In light of the notably low base editing efficiency of BE1 in mammalian cells, quantified at a mere 0.8% to 7.7%, it is postulated by the scientists that this inefficiency may be attributed to the action of uracil DNA glycosylase (UDG) (Komor et al., 2016). UDG is theorized to identify and act upon the intermediate UG base pair, restoring it to its original CG configuration through the mechanism of the base excision repair pathway (Kunz et al., 2009; Komor et al., 2016). This hypothesized intervention by UDG could be the underlying cause for the significant reduction in base editing efficiency observed within cellular environments. David Liu and colleagues ingeniously integrated a uracil glycosylase inhibitor (UGI) to the C-terminus of BE1 to create a second-generation base editor, BE2 (rAPOBEC1-XTEN-dCas9-UGI), to inhibit the base excision repair pathway and improve the editing efficiency of the cytosine editor (Komor et al., 2016). BE2 demonstrated high editing efficiency in human cells, up to 20%, which was 3-fold higher than BE1. To overcome the theoretical limitation of BE2, which is constrained by its exclusive modification of the C base in the CG base pair, limiting the maximum theoretical C-to-T conversion efficiency to 50%, researchers developed the third-generation base editor BE3 (rAPOBEC-XTEN-nCas9-UGI), substituting dCas9 with nCas9 (Komor et al., 2016). This advancement led to a significantly enhanced editing efficiency in cutting the non-edited strand of BE3, 2–6 times greater than BE2, achieving an impressive editing efficiency of approximately 37%.

#### 2.1.1 Optimizing the purity of editing products

In BE3, unintended by-products such as the conversion of targeted CG base pairs to GC or AT, along with occasional indels, have been noted (Komor et al., 2016). This is attributed to the excision of U by UDG, resulting in the formation of apurinic/apyrimidinic (AP) site that can further induce NHEJ repair and translesion synthesis (TLS), resulting in indels and C-to-A or C-to-G substitutions (Krokan and Bjørås, 2013; Wang L. et al., 2017). To improve upon this, Wang and colleagues developed an enhanced version of the base editor, termed enhanced BE (eBE) (Wang L. et al., 2017). They used one or three copies of the 2A-UGI sequence (EBE-S1 or EBE-S3) and co-expressed BE3. This modification showed lower indel frequencies and reduced generation of C-to-A/C-to-G substitutions than the original BE3 while allowing higher frequency C-to-T editing. Further advancement was made by David Liu and colleagues with the creation of the fourth-generation cytosine base editor BE4 (rAPOBEC1-XTEN-nCas9-2UGI) (Komor et al., 2017). BE4, developed by attaching a second UGI to the C-terminus of the optimized BE3 editor, showed improved C-to-T editing efficiency, reduced formation of non-T products, and decreased indel frequencies. In subsequent developments, they made further modifications to create SaBE4 and SaBE4-Gam. SaBE4 involved replacing the pyogenic *Streptococcus* Cas9n(D10A) in BE4 with *Staphylococcus aureus* Cas9n(D10A). SaBE4-Gam and BE4-Gam included an additional attachment of the Gam protein from the Mu phage, which significantly reduced indel frequency and increased product purity (Komor et al., 2017). The high-fidelity base editor (HF-BE3) represents another significant advancement (Rees et al., 2017). Employing a high-fidelity SpCas9 variant (HF-Cas9) (Kleinstiver et al., 2016) in the development of HF-BE3, based on the BE3 framework, can significantly reduce the levels of off-target editing. Furthermore, the BE-PLUS editor, developed using the SunTag system, demonstrates reduced occurrence of indels and other undesirable base substitutions compared to BE3 (Jiang et al., 2018). This enhancement not only heightens the precision of the editing process but also broadens the spectrum of edits.

#### 2.1.2 Enhancing editing efficiency and activity

Enhancing the editing efficiency and activity of base editors is a key area of focus in advancing genome editing technologies, as it directly impacts the effectiveness of these tools in inducing desired genetic modifications. The expression level of base editors within cells is particularly critical in determining their efficiency. David Liu and his colleagues developed BE4max and AncBE4max by modifying nuclear localization signals (NLS), optimizing codons, and enhancing deaminase components, based on BE4, significantly improving the efficiency of base editors across various types of mammalian cells (Koblan et al., 2018). Editors possessing higher activity can more efficiently induce the intended base changes at specific DNA targets, thereby enhancing the overall efficiency of editing. Zhang and colleagues significantly enhanced the editing efficacy of BE4max, A3A-BE4max, and eA3A-BE4max by developing hyCBE (Zhang et al., 2020a). This innovation involved integrating the ssDNA binding domain of Rad51, a crucial protein in DNA repair, between the cytidine deaminase and Cas9n components.

#### 2.1.3 Broadening the targeting scope

The PAM site plays a critical role in the deaminase-targeted editing process within CBE, with various types of Cas proteins exhibiting distinct PAM preferences. This specificity is vital for precise editing of the target genome while minimizing alterations in non-target regions. BE3, a commonly used base editor, utilizes an NGG PAM site and positions the target C within a 5 nt window close to the PAM end of the protospacer, which restricts the targeting range of CBE within the genome (Komor et al., 2016). To overcome this limitation, scientists have engineered diverse Cas proteins or their variants to recognize different PAM sequences, thereby expanding the potential editing sites in the genome using the CBE system. They have generated diverse pyrimidine base editors, effectively expanding the target range of base editing, through the utilization of different SpCas9 variants, including VQR (NGAN PAM), EQR (NGAG PAM), VRER (NGCG PAM) (Kim et al., 2017), SpCas9-NG (NG PAM) (Nishimasu et al., 2018), xCas9 (NG, GAA, and GAT PAM) (Hu et al., 2018), and SpRY (NRN > NYN PAM) (Walton et al., 2020). Additionally, integrating various Cas homologs and related variants with unique PAM specificities, like SaCas9 (NNGRRT PAM), SaKKH-Cas9 (NNNRRT PAM) (Kim et al., 2017), ScCas9 (NNG PAM) (Chatterjee et al., 2018), Cpf1 (TTTV PAM) (Li et al., 2018), and Nme2Cas9 (N4CC PAM) (Edraki et al., 2019), with base editing systems can further enhance the accuracy of base editing and broaden the genomic targeting range.

#### 2.1.4 Altering the editing activity window

The editing window is the particular region within a DNA or RNA sequence where a base editor can efficiently function and induce base changes. Optimizing this editing window to achieve highly specific editing is one of the key considerations in experimental design. Smaller windows increase specificity, allowing for precise modifications of specific nucleobases, like C, within a targeted genetic sequence. To achieve more specific editing, various base editors, including YE1-BE3, YE2-BE3, EE-BE3, and YEE-BE3, have been engineered with mutations in their cytidine deaminase domains (Kim et al., 2017). These modifications have successfully narrowed the typical 5 nt editing window to a more precise range of 1–2 nt. Further innovations, such as BE-PAPAPAP and nCDA1-BE3, which involve designing specific linker sequences and truncating the CDA1 structural domain, respectively, effectively narrow the editing activity window to 1–2 nt while maintaining high editing efficiency (Tan et al., 2019). Similarly, YFE-BE4max, created by crafting and enhancing the deaminase domain, achieves enhanced editing efficacy within a 3 nt window (Liu et al., 2020a). Conversely, for broader genomic alterations and functional regulation, a larger editing window is advantageous. The BE-PLUS editor, utilizing the SunTag system, extends the editing window from BE3’s 5 nt to 13 nt (Jiang et al., 2018). The hA3A-BE3 editor, developed based on human APOBEC3A, extends the editing window to 12 nt (Wang et al., 2018). In the realm of plant genetics, the development of the A3A-PBE plant base editor exemplifies this approach. By replacing rat APOBEC1 with human APOBEC3A on the nCas9-PBE platform and optimizing codon usage for cereal plants, A3A-PBE achieves an extended deamination window of up to 17 nt, demonstrating the versatility and adaptability of base editing systems in different contexts (Zong et al., 2018).

#### 2.1.5 Overcoming sequence context dependency

The BE3 editing construct, resulting from the fusion of rat APOBEC1, exhibits an inherent preference for specific sequence contexts and demonstrates constrained efficiency in editing GC-rich sequences (Komor et al., 2016; Gehrke et al., 2018). Recognizing the need for more versatile editing tools, David Liu et al. replaced APOBEC1 with alternative deaminases like CDA1, AID, or APOBEC3G, resulting in CDA1-BE3, AID-BE3, and APOBEC3G-BE3 (Komor et al., 2017). The CDA1-BE3 and AID-BE3 exhibited enhanced efficiency in editing cytosines following guanines compared to BE3, while APOBEC3G demonstrated a reduced sequence preference. Furthering these advancements, David Liu et al. developed a phage-assisted continuous evolutionary base editing system (BE-PACE), leading to the creation of evoAPOBEC1-BE4max and evoEFRNY-BE4max (Thuronyi et al., 2019). The evoAPOBEC1-BE4max demonstrates an increased efficiency in editing cytosine within a GC context (26 times greater compared to traditional APOBEC1), while the concurrently evolved deaminase, evoEFRNY-BE4max, consistently demonstrated high editing efficiency across all tested sequence contexts. Gehrke and colleagues used an engineered human APOBEC3A (eA3A) domain to create eA3A-BE3, which edits cytosines within the TC motif while reducing edits in other sequences (Gehrke et al., 2018). Lee and colleagues replaced the rAPOBEC1 deaminase in BE4max with an optimized human APOBEC3G variant, leading to the development of A3G-BE4.4, A3G-BE5.13, and A3G-BE5.14 (Lee et al., 2020). These editors are particularly effective and precise in CC sequence contexts. The eA3G-BE, incorporating human APOBEC3G, is tailored to edit CC sequences with a marked reduction in bystander mutations (Liu et al., 2020b). Overall, these advancements emphasize that the effectiveness of CBE editing is intricately linked to the recognition sequences of the deaminase, sequence context dependencies, and the structure of sgRNA design.

### 2.2 Adenine base editors (ABEs)

In human pathogenic point mutations, the majority involve the conversion of CG to TA base pairs, representing almost half of all mutations, while only about 14% are AT to GC mutations (Gaudelli et al., 2017). This predominance highlights the immense potential of developing base editors capable of converting AT to GC pairs. In 2017, David Liu’s laboratory made a significant advancement in this realm by developing adenine base editors (ABEs) using protein evolution and engineering techniques (Gaudelli et al., 2017). These ABEs can effectively convert AT base pairs to GC pairs within genomic DNA without necessitating DNA cleavage. They are particularly promising for correcting a wide range of single nucleotide polymorphisms related to human diseases. Notably, ABEs demonstrate superior editing efficacy and a diminished incidence of nonspecific genomic alterations, compared to the earlier base editor BE3, marking a significant leap in the performance and application of base editing technology.

The core components of ABE consist of nCas9 and a synthetically evolved adenosine deaminase, enabling the direct conversion of AT base pairs to GC base pairs (Figure 2B) (Gaudelli et al., 2017). ABE functions by integrating nCas9 with adenine deaminase, which is directed by sgRNA to target specific genomic DNA sites. During the enzymatic process, the adenine deaminase acts on ssDNA, converting A to inosine (I) within a specific range. This inosine is subsequently read or replicated as G by a polymerase, culminating in the direct transformation of AT base pairs into GC base pairs. A pivotal challenge in developing ABE was the innate limitation of natural adenine deaminases, which typically modify RNA rather than ssDNA (Harris et al., 2002). Then, Researchers selected TadA, an adenine deaminase from *Escherichia coli*, for extensive evolutionary engineering. This process led to the creation of a modified enzyme with the novel ability to act on ssDNA. The resultant ABE system, named ABE7.10, incorporates this engineered enzyme (ecTadA-ecTadA*-nCas9) and demonstrating higher efficiency and broader applicability within human cells (Gaudelli et al., 2017). The ABE7.10 system features an editing window spanning positions 4 to 9 of the sgRNA. In human cells, it achieves an impressive editing efficiency of approximately 58%, with product purity reaching 99%. A notable feature of ABE7.10 is its exceptionally low off-target activity, reported to be under 0.1%, indicating high precision and specificity in genomic editing. These characteristics of ABE, particularly its high efficiency and specificity, underscore its potential as a transformative tool in genetic research and therapeutic applications, expanding the horizons of genomic engineering.

The development of the ABE system, particularly ABE7.10 and its evolved versions, has significantly advanced the field of genomic engineering in both mammalian and non-mammalian organisms, including plants. ABE7.10 is recognized for its precise base replacement capability and minimal indels, thus optimizing this tool focuses on improving editing efficiency, expanding its activity window, and broadening its genomic editing scope. One significant advancement in this regard is ABEmax, which outperforms ABE7.10 in terms of editing efficiency (Koblan et al., 2018). ABEmax incorporates varying amounts of NLSs and optimized codon sequences, leading to a substantial boost in the editing capabilities of ABE7.10. Further developments led to CP1012-ABEmax, CP1028-ABEmax, CP1041-ABEmax, and CP1249-ABEmax, created by modifying CP-Cas9 endonuclease with bis-bpNLS and codon optimization (Huang et al., 2019; Oakes et al., 2019). These variants not only maintain the editing efficiency equivalent to ABEmax, but also expand the editing activity window from positions 4 to 9 to positions 4 to 12. While the CP-ABEmax variants expanded the editing window of ABE, many therapeutic targets could benefit from a more active ABE. ABE8, an evolution of ABE7.10 developed through an adenine deaminase variant library, displays significantly enhanced activity, achieving editing levels 1.94 times higher than those of ABE7.10 (Gaudelli et al., 2020). This increased activity makes ABE8 particularly useful for therapeutic targets that require more active base editing. Moreover, ABE8e represents a further enhancement, offering increased compatibility with Cas structural domains and heightened activity (Richter et al., 2020). Developed through phage-assisted non-continuous and continuous evolution, ABE8e features eight additional mutations that increase deamination kinetics by 590 times. It also improves expression in regions poorly edited by ABE7.10, like the BCL11A enhancer or HBG promoter, thus amplifying the effectiveness and scope of adenine base editing (Richter et al., 2020).

Despite these advancements, ABE8 variants such as ABE8e and ABE8s (Gaudelli et al., 2020; Richter et al., 2020), developed through molecular evolution, have shown a propensity for causing unintended alterations, leading to bystander mutations and off-target editing (Kim et al., 2019; Jeong et al., 2021). Addressing this, researchers developed ABE9, a precise and safe adenine base editor (Chen et al., 2023). ABE9 narrows the editing window to 1–2 nt and almost completely eliminates off-target editing on cytosine. Remarkably, ABE9 shows negligible off-target effects at both DNA and RNA levels, effectively addressing the off-target risks associated with traditional ABE systems and enhancing the safety and precision of adenine base editing (Chen et al., 2023).

### 2.3 Other types of ABEs and CBEs

#### 2.3.1 C-to-G and C-to-A transversions base editors

The widely used ABE and CBE primarily facilitate transitions between A-to-G and C-to-T bases, respectively, achieving purine-to-purine and pyrimidine-to-pyrimidine transformations. Progressing further, researchers have developed novel base editors capable of interconverting bases across different categories. Zhao et al. introduced the glycosylase base editor (GBE), an innovative adaptation of CBE, enabling C-to-G and C-to-A conversions (pyrimidine-to-purine) (Zhao et al., 2021). During the course of CBE activity, the conversion of C to U may be negated by the endogenous uracil-N-glycosylase (Ung) within the cellular milieu, resulting in an AP state that initiates the base excision repair mechanism (Komor et al., 2016; Nishida et al., 2016; Zhao et al., 2021). While CBE employs UGI to inhibit Ung activity and enhance C-to-T transition efficiency, GBE capitalizes on the AP state to facilitate conversion into other bases (Zhao et al., 2021). GBE (APOBEC-nCas9-UNG), comprised of nCas9, cytidine deaminase AID, and Ung fusion, exhibits a specific C-to-A conversion efficiency of 87.2% ± 6.9% in *Escherichia coli* and achieves specific C-to-G conversions at the sixth pyrimidine position in the N20 sequence in mammalian cells, with editing efficiency ranging from 5.3% to 53.0% (Zhao et al., 2021). To enhance the editing efficiency of GBE, researchers replaced UNG with UNG1 derived from yeast, resulting in the creation of APOBEC-nCas9-Ung1 (Sun et al., 2022). This variant showed an increase in C-to-G editing efficiency, although the purity of C-to-G conversion decreased. To further improve the C-to-G editing efficiency and purity of APOBEC-nCas9-Ung1, researchers developed APOBEC(R33A)-nCas9-Rad51-Ung1, also known as GBE2.0 (Sun et al., 2022). Compared to GBE, GBE2.0 achieves high-efficiency and high-purity C-to-G editing in mammalian cells, presenting a promising avenue for treating pathogenic G/C mutations. Another important development is the creation by researchers of C-to-G base editors (CGBEs) (Koblan et al., 2021a; Chen et al., 2021; Kurt et al., 2021). For example, CGBE1, developed by Kurt and colleagues, effectively induces C to G editing, particularly in AT-rich sequence contexts in human cells (Kurt et al., 2021). Meanwhile, the CGBEs constructed by Chen and colleagues show the greatest advantage in WCW, ACC, or GCT (W is either A or T) sequence contexts (Chen et al., 2021).

#### 2.3.2 A-to-Y transversions (where Y = C or T) base editors

The minimal off-target editing in ABE is due to the absence of an enzyme in the cell capable of efficiently excising the hypoxanthine base derived from the deamination of adenine to produce I, where I formed from A deamination is directly interpreted as G, resulting in high-purity A-to-G editing (Lau et al., 1998; Tong et al., 2023b). To achieve adenine transversion, Tong et al. fused the optimized hypoxanthine excision protein N-methylpurine DNA glycosylase (MPG) to the C-terminus of ABE8e to develop an efficient AYBE (AYBE, Y = C or T) (Tong et al., 2023b). AYBE enables A-to-C and A-to-T base editing in mammalian cells, with the optimized AYBEv3 variant achieving up to 72% conversion editing efficiency (with individual A-to-C substitution efficiency reaching up to 53% and purity up to 70%) (Tong et al., 2023b). AYBEv3 also produces fewer bystander edits compared to ABE8e and demonstrates efficient adenine transversion editing across different mammalian cell types. The advent of AYBE marks a significant advancement in base editing technology, addressing the previous inability of base editors to efficiently execute A-to-C or A-to-T changes. However, further enhancements in its editing efficiency and purity are areas for ongoing improvement.

#### 2.3.3 G-to-Y transversions base editors

Both GBE and AYBE require deamination of A or C as the initial step to trigger subsequent DNA repair processes, which limits their ability to directly edit G or T. The recent development by Tong and colleagues, a deaminase-free glycosylase-based guanine base editor (gGBE), represents a major leap in overcoming this barrier (Figure 2B) (Tong et al., 2023a). This innovative approach centers around the strategic optimization of the MPG (O'Brien and Ellenberger, 2004). The optimized MPG variants were then fused to nCas9 to create the gBE series of base editors. Within this series, the MPGv3 variant was further developed into gGBEv6.3, markedly enhancing G editing efficiency. gGBEv6.3 demonstrated remarkable guanine editing efficiency, up to 81.2% in the human genome, and maintained a low risk of off-target effects. This editor also exhibited high editing activity in mouse embryos, showcasing its potential for gene therapy and disease model development (Tong et al., 2023a). This development not only addresses the gap in direct G editing technology but also expands the scope of potential applications for genome editing.

#### 2.3.4 Dual base editors

Dual base editors are capable of simultaneously introducing changes from C to T and A to G in both plant and mammalian cells, thereby increasing the potential for mutations and possible alterations in amino acids (Grünewald et al., 2020; Li et al., 2020; Sakata et al., 2020; Xie et al., 2020). Zhang and colleagues developed the A&C BEmax, a dual base editor, by fusing cytosine deaminase (hAID) and adenine deaminase (ecTadA-ecTadA*) with nCas9, enabling it to efficiently perform concurrent A-to-G and C-to-T edits (Zhang et al., 2020b). Grünewald and colleagues combined conventional CBE and ABE approaches, positioning AID (the pivotal constituent for A-to-G alterations) at nCas9’s N-terminus and incorporating rAPO1 (the key factor for C-to-T modifications) with UGI at the C-terminus of nCas9, forming a dual base editor named SPACE (Grünewald et al., 2020). The C-T editing efficiency of SPACE is comparable to CBE, with a slightly lower A-G editing efficiency. However, the efficiency of the dual-base editor SPACE surpasses that of CBE + ABE. Li et al. created STEME, a saturated dual-base editing tool targeting endogenous gene mutations in plants (Li et al., 2020). STEME-1 exhibited high C-to-T induction efficiency up to 61.61% in rice protoplasts and achieved 15.50% efficiency for simultaneous C-to-T and A-to-G mutations. Zhang and colleagues significantly enhanced the efficiency of the dual-base editor by fusing the deaminases evoFERNY and TadA8e at the N-terminus of nCas9-NG, and by attaching two UGIs at the C-terminus to create STCBE-2 (Zhang et al., 2023b). Additionally, researchers developed the dual-base editors Target-ACEmax (Sakata et al., 2020) and ACBE (Xie et al., 2020), achieving C-to-T and A-to-G conversions in mammalian systems, while AGBE (Liang et al., 2022) can induce four types of base alterations (C-to-G, C-to-T, C-to-A, and A-to-G). Though dual-base gene editing tools still require further research and refinement, they represent a more expedient and efficient approach to genetic editing, providing a new perspective for the ongoing advancement and application of base editing tools.

## 3 Base editing in RNA

**FIGURE 3:**
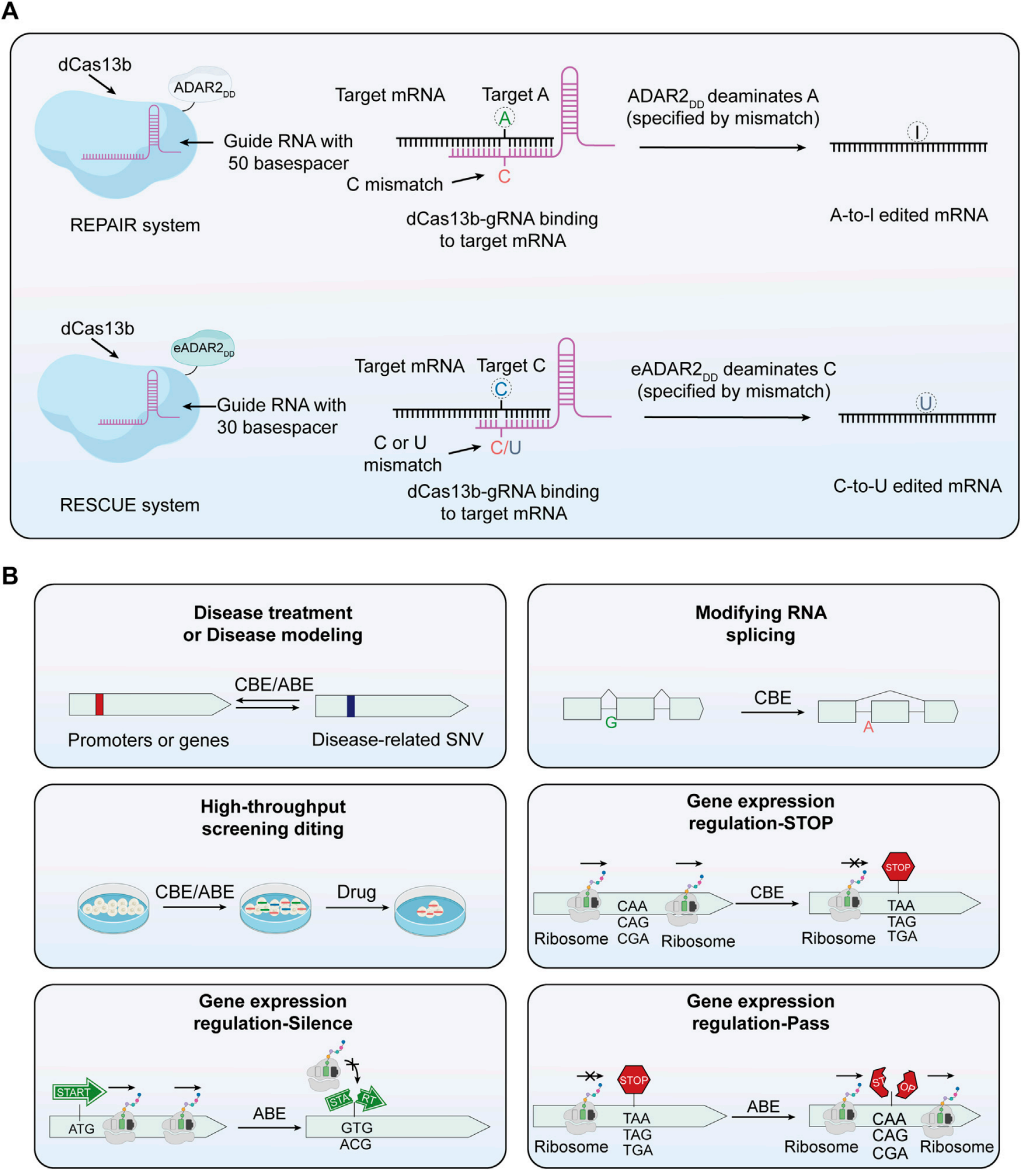
The principle of RNA base editors and the multifunctionality of base editors. **(A)** Mechanism of RNA base editors. This section expounds the mechanism of RNA base editing, achieving A-to-I and A-to-U edits. The REPAIR system employs a fusion of dCas13 with the catalytic domain of ADAR deaminase to facilitate programmable A-to-I replacement. Through specifically designed guide RNAs (gRNAs), the dCas13-ADAR complex is directed to precise sites on RNA molecules. In this context, capitalizing on the induced AC mismatch between the target mRNA and the gRNA of Cas13b, the catalytic domain of ADAR subsequently converts A at the targeted site into I. In the RESCUE system, an enhanced version of ADAR2 can convert C to U. Leveraging induced CC or CU mismatches between the target mRNA and the gRNA of Cas13b, the ADAR2 variant achieves targeted deamination of cytosine on mRNA. **(B)** The multifunctionality of base editors. Base editing technology, in addition to correcting mutated genes, can be used for a variety of other applications. These include editing RNA splicing receptors, conducting functional screening of single nucleotide variants, and regulating gene expression, among others.

The foremost advantage of RNA editing over DNA editing is its reversible nature, stemming from the inherently short lifespan of RNA. This attribute enhances safety by allowing modifications to be temporary. Particularly in cases of diseases arising from abnormal transcript splicing, where DNA editing might not be effective, RNA editing becomes crucial for successful treatment. Consequently, RNA editing not only complements DNA editing across various domains but also offers unique and significant advantages in specific areas. The origins of RNA base editing trace back to 1995, when scientists began pioneering methods to mimic substrates for adenosine deaminases acting on RNA (ADAR) by designing complementary RNA strands, thereby enabling targeted editing at specific RNA sites (Woolf et al., 1995). ADAR enzymes, which catalyze the conversion of A to I, play a crucial role in RNA editing mechanisms and are also a significant component of epigenetic regulation (O'Connell et al., 1998; Nishikura, 2010). Subsequent advancements included the engineering of deaminase expression and chemically modified guideRNAs, markedly enhancing the specificity and efficiency of RNA editing (Montiel-Gonzalez et al., 2013; Vogel et al., 2014). Techniques utilizing endogenous ADAR proteins for editing have also emerged, exemplified by the trans-acting guideRNA designed by Wettengel et al. and the LEAPER system established by Qu et al. (Wettengel et al., 2017; Qu et al., 2019). These advancements markedly augment the recruitment capabilities of ADAR and enhance the precision of RNA editing. A major breakthrough in this field emerged with the introduction of RNA base editing techniques based on the CRISPR/Cas system. This technology harnesses the targeting capability of the CRISPR/Cas system and specific Cas proteins, like Cas13, for precise RNA base substitution (Cox et al., 2017; Wolter and Puchta, 2018). Cas13 is distinguished by its highly conserved HEPN nucleic acid-binding domain, allowing for the binding and cleavage of RNA targets, and its specificity for the protospacer flanking site, which leads to a preference for cleaving targets with protospacer flanking site (Abudayyeh et al., 2016). Cox et al. utilized dCas13b (lacking nuclease activity but retaining binding capacity) fused with ADAR deaminase to construct REPAIRv1, achieving programmable A to I substitution (Figure 3A) (Cox et al., 2017). To enhance the specificity of REPAIRv1, researchers introduced point mutations into the ADAR deaminase domain, leading to the development of REPAIRv2. This modification significantly reduced off-target editing. Building on the REPAIR technology, Abudayyeh and colleagues further modified the active domain of the ADAR2 enzyme to develop RESCUE (RNA editing for specific C to U exchange), an RNA editor with dual A-I and C-U deaminase capabilities (Figure 3A) (Abudayyeh et al., 2019). Undoubtedly, the dual substrate deaminase capability of RESCUE expands its range of potential applications.

The large size of the Cas13 protein, particularly when fused with a deaminase domain, presents a challenge for its encapsulation into a single adeno-associated virus for efficient *in vivo* delivery, thereby constraining its application in *in vivo* therapies. To address this, developing more compact RNA base editors that maintain high editing efficiency and can be packaged into a single AAV vector is crucial for their widespread application. Kannan et al. discovered an ultra-compact Cas13b enzyme, Cas13bt, consisting of 775–804 amino acids (aa), from a pool of thousands of Cas13 enzymes (Kannan et al., 2022). This enzyme was incorporated into REPAIR and RESCUE systems to create RNA base editors capable of facilitating A-I mutations (REPAIR.t1 and REPAIR. t3) and C-U mutations (RESCUE.t1 and RESCUE. t3) (Kannan et al., 2022). Xu et al. identified two compact families of CRISPR-Cas ribonucleases (775–803 aa) from high-salt samples, denoted as Cas13X and Cas13Y (Xu C. et al., 2021). They combined dCas13X.1 with a high-fidelity ADAR2dd and an evolutionarily-derived ADAR2 deaminase to create the A-to-I RNA base editor xABE and the C-to-U editor xCBE (Xu C. et al., 2021). Both editors demonstrated high editing efficiency and specificity. Recently, Wang et al. developed a more compact RNA base editor (ceRBE) by replacing the larger dCas13 protein with a smaller 199-amino acid EcCas6e protein and fusing it with the ADAR deaminase (Wang et al., 2023c). When delivered to DMD mice via a single AAV, ceRBE achieved an *in vivo* editing efficiency of 68.3% ± 10.1%, presenting a promising RNA-based approach for treating genetic diseases. These advancements signify a significant step toward the practical application of RNA base editing in therapeutic contexts.

## 4 Delivery strategies

The advent of therapeutic gene editing within the human body marks a significant milestone, with the base conversion capabilities of base editors offering vast potential and broad applicability in this field. These tools exhibit vast potential and diverse applicability, transcending mere correction of gene mutations (Figure 3B). For instance, base editors can be utilized to edit RNA splicing acceptors, which is of significant importance for understanding and treating diseases related to RNA splicing (Gapinske et al., 2018; Jeong et al., 2020). They also facilitate rapid functional screening of single nucleotide variants, deepening our understanding of genetic mutations (Kweon et al., 2020; Hanna et al., 2021). Furthermore, the use of base editors in the regulation of gene expression opens up new opportunities for precision medicine and personalized treatment approaches (Jeong et al., 2020). By modulating gene expression, these tools can be tailored to individual patient needs, offering more targeted therapeutic interventions. The effectiveness of gene editing tools, including base editors, is heavily dependent on delivery strategies. These editors can be introduced into cells through various means, including DNA that encodes their expression, as mRNA, or directly in the form of proteins and ribonucleoproteins (RNPs) (Raguram et al., 2022). DNA vectors, often plasmids, carry sequences encoding the editing tools. Once inside the cell nucleus, these sequences are transcribed and translated into functional editing machinery. In contrast, mRNA delivery, a non-traditional approach, eliminates the need for DNA transcription, enabling rapid translation into editing proteins. Protein and RNP forms offer an even more direct approach, bypassing both transcription and translation stages, and directly engage in editing within cells. Each delivery method has unique attributes, and the choice of method should be based on factors like editing efficiency, speed, expression levels, and the specific requirements of the application. This range of choices provides flexibility and adaptability for gene editing research, driving ongoing improvements and innovations in delivery techniques.

### 4.1 Viral vector delivery methods

**FIGURE 4:**
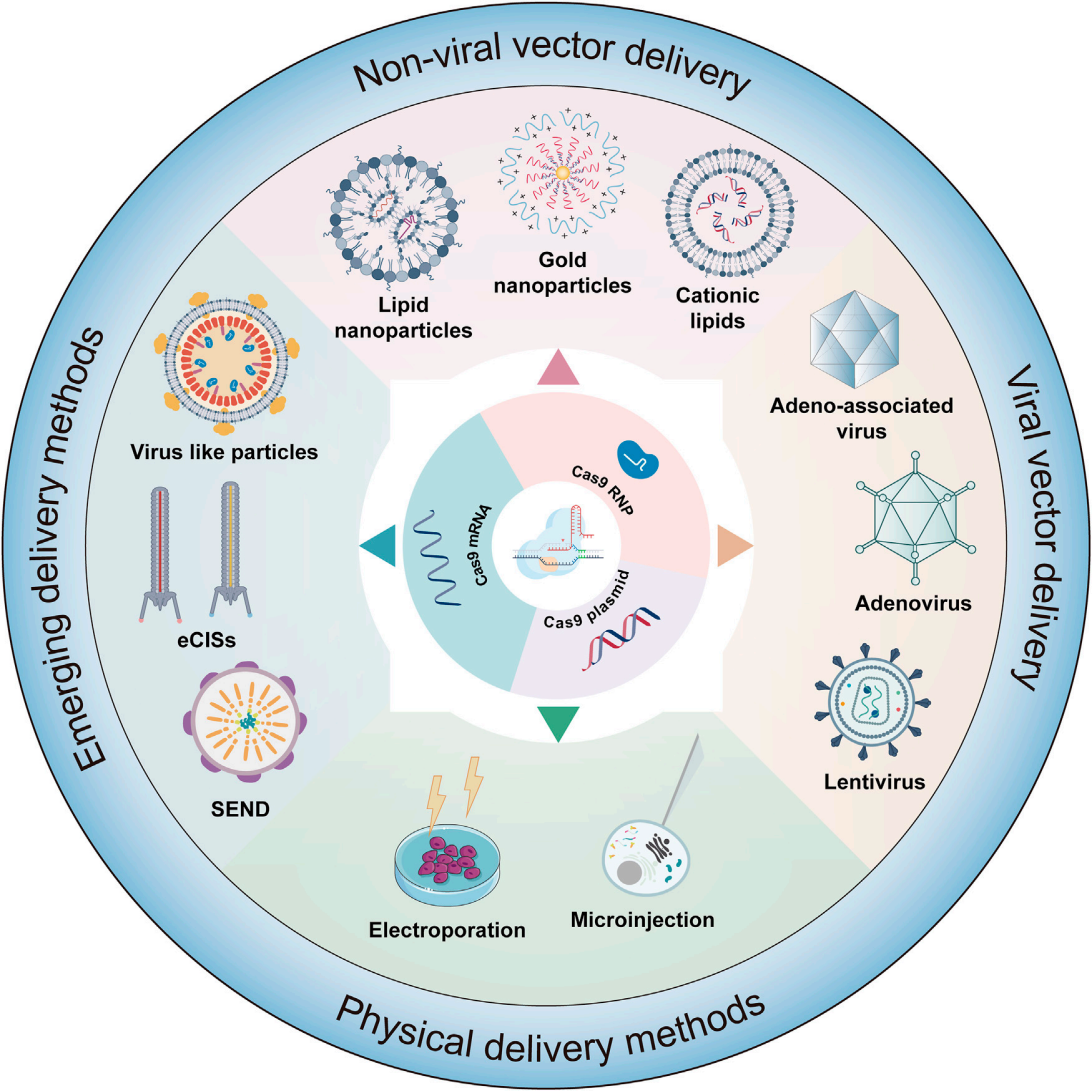
Delivery strategies of gene editing systems. This figure details the various methodologies employed in the delivery of editors in forms such as mRNA, plasmids, or ribonucleoprotein (RNP) complexes. The delivery mechanisms are classified into several categories: viral vector delivery, physical delivery method, nonviral delivery, and emerging delivery strategies.

Currently, gene therapy primarily utilizes two delivery forms: viral and nonviral (Figure 4). Among these, viral vectors are a popular choice for *in vivo* gene editing, with adeno-associated virus (AAV), lentivirus (LV), and adenovirus (Ad) being the most common. These carriers are engineered to transport editing tools and necessary components into various tissues or organs through injection or targeted delivery. Viral vectors offer advantages like high efficiency, specificity, and the ability to deliver across a myriad of cell types and tissues. AAV is particularly notable for its versatility and safety in gene therapy applications (Table 1). They consist of a protein capsid enclosing a 4.7 kb ssDNA genome and are non-pathogenic (Wang et al., 2019). Their benefits include high delivery efficiency, low immunogenicity, broad cell-transducing capabilities, and stable, long-term expression (Wang et al., 2019). AAV is versatile in its delivery to various tissues, such as the liver, central nervous system, muscles, and ocular tissues (Naso et al., 2017; Wang et al., 2019). Clinically, AAV has a solid track record and has been extensively used in gene therapy trials (Ail et al., 2023; Muramatsu and Muramatsu, 2023). However, delivering large base editors via AAV presents challenges due to size constraints. To overcome AAV packaging limitations, researchers have developed a dual AAV strategy. This involves two separate AAV vectors, each carrying parts of the editing tools and components, which reassemble into a functional editing enzyme or protein through intein-mediated trans-splicing within the target cells (Truong et al., 2015). It has been reported that multiple groups have utilized a dual AAV system to deliver base editing treatments for diseases such as amyotrophic lateral sclerosis (Lim et al., 2020), duchenne muscular dystrophy (DMD) (Ryu et al., 2018; Xu L. et al., 2021; Chemello et al., 2021), metabolic liver diseases (Villiger et al., 2018), hutchinson-gilford progeria syndrome (Koblan et al., 2021b), and hearing loss (HL) (Yeh et al., 2020) in mouse models. In addition, researchers have developed smaller Cas nucleases to enable single AAV vector delivery (Chen et al., 2022; Kweon et al., 2023) and strategies to reduce the long-term expression of edited genes via AAV delivery (Ibraheim et al., 2021; Zhang H. et al., 2023). Despite these advancements, the clinical application of AAV vectors still faces certain limitations, with pre-existing immunity against AAV being a major challenge in AAV gene therapy (Kruzik et al., 2019).

**TABLE 1:** An overview and comparison of viral delivery methods in gene editing.

Vector	Packaging capacity (kb)	Diameter (nm)	Summary of technical aspects	Advantages	Challenges	Refs
Adeno-associated virus	∼4.7	20–25	ssDNA; Entering the cell through endocytosis; Broad range of target cells dependent on the serotype	Long-term expression; Having multiple serotypes; Low integration of vector genome sequences; Low immunogenicity	Payload capacity limitation; Prolonged expression may lead to an increased off-target probability; Pre-existing immunity	Naso et al. (2017), Wang et al. (2019)
Lentivirus	∼10	80–120	ssRNA; Entering cells through membrane fusion or endocytosis; Broad range of target cells dependent on the envelope	Large payload; Highly efficient and stable efficiency; No pre-existing immunogenicity	Immunogenicity; Potential genome integration	Ferreira et al. (2021), Wang et al. (2021)
Adenovirus	8–36	90–100	dsDNA; Entering cells through endocytosis; Broad range of target cells	High payload capacity; High efficiency; Non-integrative into the genome	Immunogenicity; Pre-existing Immunity	Lee et al. (2017a), Ortinski et al. (2017)

The majority of *in vivo* gene editing applications have harnessed AAV, whereas certain preclinical investigations have availed themselves of lentiviruses or adenoviruses. Lentiviruses, derived from the human immunodeficiency virus (HIV), differ from AAV in their ability to carry larger genetic payloads and, in certain cases, achieve higher delivery efficiency (Ferreira et al., 2021). Integrating the editing tools into the lentivirus genome enables its direct injection or targeted delivery into tissues or organs in the body. For example, lentivirus and integration-deficient lentiviral vectors (IDLVs) have been used to deliver ABEmax for correcting the RPE65 pathogenic gene in the retinal pigment epithelium of mice (Suh et al., 2021). Ortinski and colleagues demonstrated efficient genome editing using IDLVs in HEK293T cells and post-mitotic brain neurons (Ortinski et al., 2017). However, a significant drawback of *in vivo* lentiviral delivery is the potential for genome integration (Kymäläinen et al., 2014). Adenoviruses, on the other hand, are non-enveloped DNA viruses that infect a wide range of host cells with high efficiency (Lee C. S. et al., 2017). Research has shown that adenovirus-mediated base editors introduce precise nucleotide changes in cells and tissues of mammals (Chadwick et al., 2017; Carreras et al., 2019). While Ad facilitates potent gene editing, it bears the potential to induce the production of antibodies against Cas9 (Wang et al., 2015).

Viral vectors are essential delivery tools in gene therapy and genetic editing, with current challenges focusing on delivery efficiency, specificity, and the potential for triggering immune responses, especially in *in vivo* applications. Future research is aimed at improving these vectors to enhance delivery efficiency and specificity while minimizing immune responses, making them safer and more effective. Additionally, combining viral vectors with other delivery methods or employing genome editing to optimize viral vectors themselves are promising areas of development.

### 4.2 Nonviral vector delivery methods

Nonviral delivery methods play a crucial role in the field of drug and gene therapy, offering a range of advantages over traditional viral vector-based methods (Table 2). One of the key benefits of nonviral delivery methods is their reduced immunogenicity. Unlike viral vectors, nonviral methods typically do not elicit strong immune responses in the body. This reduced immunogenicity is crucial for repeated administration and long-term treatments, as it minimizes the risk of the body developing resistance or adverse reactions to the therapy (Cheng H. et al., 2021; Raguram et al., 2022). Another advantage is the lower toxicity associated with nonviral delivery systems. These methods are generally considered safer and more biocompatible, reducing the risk of adverse reactions and side effects that can be associated with viral vector-based therapies.

**TABLE 2:** A summary of recently popular nonviral delivery methods.

System	Size	Summary of technical aspects	Advantages	Challenges	Refs
Electroporation	NA	DNA, mRNA, or Proteins; Physical delivery method involving disruption of the cell membrane via electrical pulses	High efficiency; Broadly applicable	May result in cellular damage or apoptosis; Requires specialized equipment and expertise	Batista Napotnik et al. (2021), Campelo et al. (2023)
Microinjection	NA	DNA, mRNA, or Proteins; Physical strategies for delivery using microneedle arrays	High precision; Broadly applicable; Highly controllable	Demands high technical proficiency in operation; Low delivery efficiency	Xu, 2019; Cheng et al. (2021a), Taha et al. (2022)
Lipid nanoparticles	150–200 nm	DNA, mRNA or RNPs; Entering cells through endocytosis	Transient expression; Low immunogenicity; High biocompatibility; Biodegradability	Poor long-term stability; Mostly ends up in the liver; Lack of organ/cell specificity	Hou et al. (2021), Wang et al. (2022a), Taha et al. (2022)
Virus-like particles	∼100 nm	Proteins or mRNA; Entry into cells via injection, electroporation, cell-penetrating peptides, extracellular vesicles, etc	Transient expression; Simple structure, amenable to engineering design; No risk of genomic integration; Minimal off-target risk	Potential immunogenicity; Potential instability	Lyu et al. (2020), He et al. (2022), Mejía-Méndez et al. (2022)
Extracellular contractile injection systems (PVC- eCIS)	80–180 nm	Proteins; Tail fiber proteins recognize and attach to the cell membrane, the outer sheath contracts, and the inner tube delivers proteins into the cell through a central spike	Extremely high efficiency; Can deliver virtually any form of protein; Preparation steps are straightforward; Low Immunogenicity	Whether it possesses advantages in both delivery efficiency and safety still requires extensive exploration	Jiang et al. (2022), Heiman et al. (2023), Kreitz et al. (2023)

Abbreviations: NA, not applicable.

#### 4.2.1 Physical delivery methods

Physical methods, such as electroporation and microinjection, directly introduce genetic material into cells, typically used in laboratory settings. Electroporation, which uses high-intensity electrical pulses to transiently increase cell membrane permeability and generate pores, allows editing tools to enter cells and is valued for its simplicity, broad applicability, and effectiveness across various cell cycle stages in almost all cell types (Cheng H. et al., 2021; Campelo et al., 2023). Electroporation has shown excellent delivery efficiency in gene editing, including CRISPR/Cas9-based embryo editing in mice and rats (Kaneko and Nakagawa, 2020; Kaneko, 2023) and primary T cell editing with BE3 and BE4 (Webber et al., 2019). Furthermore, electroporation-based CRISPR therapies for β-thalassemia and sickle cell disease (SCD) have progressed to clinical trials (Frangoul et al., 2021). However, electroporation can sometimes lead to cell damage or apoptosis in certain cell types and tissues, necessitating optimization and safety assessments for each specific application (Wang H. X. et al., 2017). Microinjection, another physical strategy, involves using micrometer-sized needles to directly deliver DNA, mRNA, or proteins into target cells. This method allows precise control and avoids non-specific editing. As a commonly used cellular injection technique, microinjection has been effectively applied in various cell and animal studies, including transgenic animals, animal cloning, early embryo editing, and nucleases-mediated DNA double-strand breaks (Xu, 2019; Cheng H. et al., 2021). Recent research has demonstrated that microinjecting CBE into porcine embryonic fibroblasts and embryos enables precise C to T editing (Song et al., 2022). The challenges of microinjection include the need for high operational precision, potential impacts on the cells during the injection process, and considerations for cell survival rates pre- and post-injection. Therefore, careful selection and adjustment of injection parameters are essential when utilizing microinjection to ensure accurate and effective experimental outcomes. The decision to use electroporation or microinjection for gene editing is influenced by factors like the specific cell type being targeted, the desired efficiency of the editing process, and the level of precision required for the particular gene editing task.

#### 4.2.2 Nanoparticle delivery methods

In the field of gene editing, the application of nanoparticles has opened new avenues for precise interventions in complex biological systems. These tiny engineered materials can effectively encapsulate and protect gene editing tools, enabling their safe delivery to target cells. In particular, lipid nanoparticles (LNPs) have shown significant potential in this area. LNPs are nanoscale particles encapsulated by a lipid bilayer, typically composed of cationic or ionizable lipids, neutral helper lipids, polyethylene glycol (PEG) lipids, and cholesterol (Jeong et al., 2023). These components collectively enable LNPs to effectively transport biologically active substances like nucleic acids and proteins into cells. The intracellular delivery efficiency of LNPs can be finely tuned through rational design and modification, by altering aspects such as lipid composition, particle size, and surface characteristics (Paunovska et al., 2022; Wang et al., 2023b). The key advantages of LNPs include their extremely small particle size, high stability, and biocompatibility, which enable effective drug protection and delivery efficiency. One of the characteristics of conventional LNPs is their resemblance to low-density lipoprotein (LDL), which leads to their recognition and uptake by LDL receptors predominantly present in liver cells (Raguram et al., 2022). This results in the accumulation of LNPs in the liver, posing a limitation for their use in targeting non-hepatic tissues or organs. To overcome this challenge and expand the utility of LNPs beyond hepatic applications, researchers have been developing various innovative strategies. One such approach involves localized LNP injections, where Palanki and colleagues have successfully identified the most effective LNPs in the perinatal mouse brain by administering intracerebroventricular (ICV) injections in fetal and neonatal mice (Palanki et al., 2023). Another strategy is the use of DNA barcoding to identify non-hepatic cell-tropic LNPs (Dahlman et al., 2017; Ni et al., 2022). This method leverages unique DNA barcodes to modify distinct LNPs, resulting in a highly selective and efficient nanodelivery system targeted towards specific cells or tissues. Targeted modification of LNPs to achieve redirection is also a promising approach. For instance, the fusion of the binding domains D1 and D2 of the cell adhesion molecule MAdCAM-1 onto IgG-Fc, establishing targeted LNPs with the high-affinity conformation of integrin α4β7 in the gut, can specifically silence (IFN-γ) in the intestinal tracts of colitis mouse models (Dammes et al., 2021). By combining the membrane-anchored lipoprotein ASSET of LNPs with the Fc region of antibodies, specificity towards different cell subgroups can be achieved through the alteration of variable regions (Kedmi et al., 2018). Pre-treatment with a Nanoprimer, which occupies liver cells and reduces their uptake of LNPs, has also been shown to enhance the delivery efficiency of RNA-based therapies (Saunders et al., 2020). The selective organ targeting (SORT) technique, which involves modulating the internal charge of LNPs, allows for precise and predictable optimization (Cheng et al., 2020). This technique facilitates the expedited and targeted delivery of diverse payloads to the pulmonary, splenic, and hepatic tissues in murine models.

The LNP delivery system, in contrast to viral delivery methods, does not involve the introduction of live viral particles. This significantly reduces the potential risks associated with immune responses and cellular toxicity. Currently, several studies have demonstrated that the use of LNPs for delivering base editors can achieve effective genome editing in both mice and primates (Musunuru et al., 2021; Rothgangl et al., 2021; Adair et al., 2023). LNPs have shown promising efficacy in both preclinical and clinical stages (Hou et al., 2021). Nonetheless, this delivery system faces certain limitations, such as variability in delivery and editing efficiency across different cell types, along with potential issues related to low immunogenicity and long-term stability (Hou et al., 2021; Taha et al., 2022). In addition to LNPs, gold nanoparticles and cationic lipid particles have also demonstrated their unique value in the delivery platforms for gene editing tools. Gold nanoparticles, existing at the nanoscale, have garnered attention in scientific research and technological applications owing to their distinctive physicochemical properties (Kumar et al., 2013). Gold nanoparticles, typically ranging from 1 to 100 nm in diameter, exhibit distinct characteristics from bulk gold materials, with their most notable feature being surface plasmon resonance, providing strong light absorption and scattering capabilities in the visible to near-infrared spectrum (Sharifi et al., 2019). Gold nanoparticles can undergo surface modification to establish stable complexes with DNA or RNA molecules, thereby assuming a crucial role in drug delivery and the transportation of gene editing agents (Giljohann et al., 2009; Lee K. et al., 2017; Yañez-Aulestia et al., 2022). On the other hand, cationic lipid particles, serving as nonviral delivery carriers, also demonstrate substantial potential in gene editing applications. These particles, due to their positive charge, establish stable complexes with negatively charged nucleic acids, thereby facilitating their cellular uptake through endocytosis (Elouahabi and Ruysschaert, 2005). This mechanism facilitates the efficient delivery of gene editors into the interior of target cells, where cationic lipid particles can release their payload through intracellular interactions, promoting the expression and function of the gene editors (Shi et al., 2016; Ponti et al., 2021).

#### 4.2.3 Virus-like particles (VLP) delivery strategies

VLPs are non-infectious particles self-assembled from viral surface proteins, possessing the structural characteristics and immunogenicity of viruses but lacking a genetic genome (Chandler et al., 2017). These characteristics make VLPs a safe and effective delivery platform, positioning them as a promising carrier for delivering gene editing agents (Lyu et al., 2020). In recent years, numerous studies have demonstrated the potential of VLPs as gene delivery systems, with a common method being the loading of editing tool mRNA into VLPs. By loading mRNA into genome-deleted or inactivated VLPs, it enables the transcription and translation processes within target cells, thereby facilitating the expression of gene editing effectors. Mock and colleagues used a retroviral vector with deactivated reverse transcriptase to deliver TALEN mRNA, effectively transducing cells and supporting transient transgene expression (Mock et al., 2014). Prel and colleagues developed MS2 chimeric RNA lentiviral particles (MS2RLPs) by optimizing the interaction between the bacteriophage MS2 capsid protein and MS2 RNA genome (Prel et al., 2015). Moreover, researchers have developed VLPs for the efficient delivery of CRISPR/Cas9 components, utilizing a range of viral capsids for enhanced targeting and delivery efficiency (Lindel et al., 2019; Ling et al., 2021; Yin et al., 2021).

The demand for transient expression of gene editing effectors has driven the development of VLP delivery strategies. A significant strategy involves packaging editing tools as proteins or RNPs within the VLPs. Choi and colleagues developed a VLP system that simultaneously expresses pre-packaged Cas9 protein and its corresponding sgRNA (Choi et al., 2016). In this system, the Cas9 sequence is fused to the N-terminus of the Gag gene, incorporating an HIV-1 protease cleavage site between them. This configuration allows the functional Cas9 protein to be released during particle maturation. Lyu and colleagues employed specific aptamer/aptamer-binding protein (ABP) interactions to package Cas9 RNPs into LV capsids, achieving highly targeted base editing and negligible RNA off-target activity (Lyu et al., 2021). David Liu and colleagues engineered DNA-free virus-like particles (eVLPs) based on a retroviral scaffold by optimizing the linker region, improving gag-cargo positioning and dosage, resulting in highly efficient base editing across various major mouse and human cell types (Banskota et al., 2022). Li et al. established a vaccine delivery system named VLP@Silica, utilizing VLPs as biological templates to self-assemble with silica, enabling the creation of a versatile nanoadjuvant vaccine, thereby presenting a novel approach to VLP-based vaccine design (Li M. et al., 2022). Segel et al. has innovatively created a novel endogenous RNA delivery platform termed selective endogenous encapsidation for cellular delivery (SEND), based on the long terminal repeat retrotransposon homolog PEG10, which achieved targeted cell RNA delivery through optimized modifications of the PEG10 protein (Segel et al., 2021). As the RNA carriers utilized by the SEND platform are derived from endogenous proteins, this suggests that the system does not elicit an immune response within the body, significantly reducing potential side effects. In the future, SEND technology may potentially replace lipid nanoparticles and viral vectors, emerging as the most suitable carrier for gene editing therapies.

#### 4.2.4 The extracellular contractile injection systems

The bacterial contractile injection systems (CIS) are crucial cellular-puncturing nanodevices, featuring an elastic structure akin to the tail of T4 bacteriophages (Taylor et al., 2018). They actively inject various cargo proteins into prokaryotic or eukaryotic cells, utilizing the stored energy in sheath proteins for this purpose. Based on differences in their mechanisms of action, bacterial CIS can be broadly categorized into the cell-based type VI secretion systems (T6SSs) and the extracellular CIS (eCIS) (Heiman et al., 2023). The Photorhabdus Virulence Cassette (PVC), a subtype of eCIS, primarily consists of an outer sheath and an inner tube, which together play an important role in the delivery and injection of effector molecules into host cells (Wang X. et al., 2022). The Jiang team has identified a class of N-terminal signal peptides capable of importing proteins from various origins into PVC and transporting them into eukaryotic cells (Jiang et al., 2022). This method has been successfully applied for targeted tumor therapy in experimental animals. Kreitz et al. has elucidated that the interaction between the tail fiber protein Pvc13 and cell membrane receptors is key for PVC to recognize target cells, addressing the targeting challenge associated with PVCs (Kreitz et al., 2023). Using the AlphaFold tool for protein engineering modifications of Pvc13 can enable efficient delivery of functional proteins at the cellular and animal levels, without inducing immunogenicity or toxicity. The recent progress in the PVC system indeed presents a promising new avenue in the field of therapeutic agent delivery, particularly for advanced gene therapies involving CRISPR-Cas9 and base editors. As research in this area continues to advance, it holds the promise of bringing more precise and effective solutions to some of the most challenging diseases facing humanity.

## 5 The application of base editing in the treatment of genetic diseases

**FIGURE 5:**
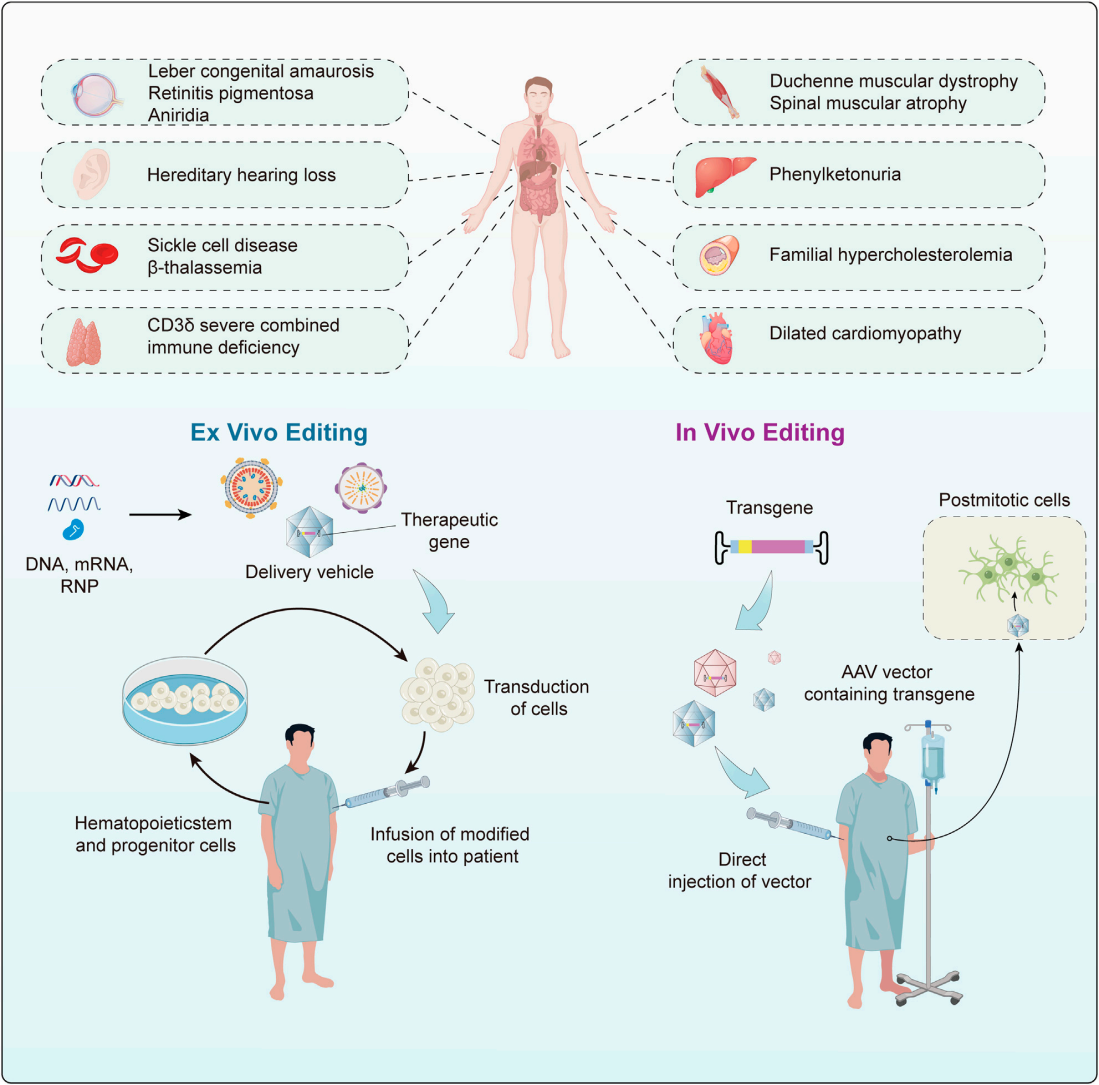
Comprehensive overview of base editing applications and strategies in genetic disease treatment. It includes an overview of major diseases that are being targeted with base editing technology, as well as the strategies employed for their treatment, both *in vivo* and *ex vivo*.

In traditional medicine, genetic diseases can generally are typically categorized into single-gene diseases, chromosomal diseases, and multifactorial diseases (Iourov et al., 2019). Common genetic diseases encompass hereditary retinal disorders, hereditary deafness, and hereditary blood disorders, among others. In the past, the main methods for treating genetic diseases involved attempts to repair genetic defects in patients through approaches like gene replacement, gene addition, or stem cell transplantation, but these methods encountered various issues, including technical complexity, precision control challenges, and high costs (Mingozzi and High, 2011; Ragni, 2021; Weber, 2021). The advent of CRISPR/Cas9 technology has revolutionized the field, offering new possibilities for treating human genetic diseases. Its precision in genome editing has made it a valuable tool in various clinical and therapeutic applications, yet its double-stranded breaks may lead to some unpredictable consequences (Qin et al., 2023a; Bhatia et al., 2023). The base editing technique enables precise modification of the human genome by achieving targeted conversion independent of HDR and double-strand DNA breaks, directly altering individual base pairs within DNA sequences. This innovation constitutes a significant advance in rectifying mutations associated with genetic disorders, positioning it as a potential groundbreaking approach for treating hereditary diseases (Figure 5). To date, base editing has been effectively employed in addressing a spectrum of genetic conditions (Table 3), heralding its potential to emerge as an important modality for the future management of hereditary diseases.

**TABLE 3:** Genetic disease treatment strategy based on base editing.

Disorder	Cells/models	Strategy	Delivery	Therapeutic outcomes	Refs
DMD	DMD mouse	ABE7.10	Intramuscular injection of tsAAV	Restored dystrophin expression in 17% ± 1% of myofibers	Ryu et al. (2018)
DMD	mdx^4cv^ mouse	iABE-NGA	Tail vein injection of dual AAV9	Over 95% of cardiac muscle cells restored dystrophin expression, improving muscle tissue pathology	Xu et al. (2021b)
DMD	DMDΔ44 myoblast cell line, C57BL/6J mouse	FNLS, ABERA, ABE8e	Intramuscular injection AAV9 or MyoAAV	*In vitro*, FNLS resulted in a 77.9% exon skipping rate, while ABERA led to a 55.0% exon skipping rate. *In vivo*, the highest editing efficiency of ABE8e was 23%	Gapinske et al. (2023)
DMD	∆Ex51 mouse	ABEmax-SpCas9-NG	Intramuscular injection of dual AAV9	96.5% ± 0.7% of muscle fibers restored expression of dystrophin	Chemello et al. (2021)
DMD	Dmd^E4*^ mouse	eTAM	Intraperitoneal injection of dual AAV9	Exon-skipping efficiency: 60.27% ± 8.05%, dystrophin: 84.0% ± 6.3% of WT levels, alleviating muscular dystrophy for at least 1 year	Li et al. (2021b)
DMD	Patient-derived iPSC-CMs	TAM	—	99.9% of DMD transcripts undergo exon 50 skipping, leading to the restoration of the open reading frame in almost all transfected cells	Yuan et al. (2018)
DMD	ΔEx48–54 iCMs	ABE8eV106W-SpCas9	Electroporation	Restored dystrophin protein expression to 42.5% ± 11% of WT levels	Wang et al. (2023a)
DMD	DMD^E30mut^ mouse	mxABE	Intramuscular injection of AAV9	Achieving an editing rate of up to 84% for A>G, sustained treatment restored dystrophic protein expression to 50% of WT levels, significantly improving muscle function	Li et al. (2023)
DMD	DMD^Q1392X^ mouse	ceRBE	Intramuscular injection of AAV9	*In vivo* editing efficiency reached 68.3% ± 10.1%, rescuing dystrophin protein in TA tissue to a high level of 68.1% ± 9.4%	Wang et al. (2023c)
LCA	rd12 mouse	ABEmax	Subretinal injection of LV	Maximum correction rate can reach up to 29%, with restoration of visual cycles, intact pathway functionality from the retina to the primary visual cortex, and recovery of cortical responses	Suh et al. (2021)
LCA	rd12 mouse	NG-ABE	Subretinal injection of dual AAV9	Restored approximately 80% of RPE65 mRNA expression, with ERG amplitude reaching 60% of WT levels	Jo et al. (2023b)
LCA	rd12 mouse	NG-ABE	Subretinal injection of LV	Rescuing 40% of functional allele genes, restoring cone cell-mediated visual function, and preserving cone cells in LCA mice	Choi et al. (2022)
LCA	rd12 mouse	NG-ABEmax	Subretinal injection of RNP	Maximum correction efficiency in juvenile mice is 5.7%	Jang et al. (2021)
LCA	*Kcnj13* ^*W53X/+ΔR*^ mouse	ABE8e	Subretinal injection of SNC	*In vivo* RPE editing efficiency of ABE8e is 16.8% ± 7.9%, restoring the ERG c-wave amplitude	Kabra et al. (2023)
RP	rd10 mouse	NG-ABE8	Subretinal injection of dual AAV8	At the cDNA level, the average correction efficiency reached as high as 54.97%, restoring PDE6B expression, preserving photoreceptors, and rescuing visual function	Su et al. (2023)
RP	rd10 mouse	SpRY-ABE8e	Subretinal injection of dual AAV5	Correction rate of PDE6B cDNA reached as high as 49%, preserving the morphology of photoreceptors and significantly improving visual function	Wu et al. (2023)
Aniridia	CHuMMMs aniridia cell lines, Sey mouse	ABE8e	Electroporation LNP	Average correction rate *in vitro* is 76.8% ± 0.48%; average editing efficiency of the Pax6 patient variant in mouse cortical neurons *ex vivo* is 2.33% ± 1.0%	Adair et al. (2023)
HL	Baringo mouse	AID-BE4max	Inner ear injection of dual AAV2	The *in vivo* editing efficiency of Tmc1 mRNA reached up to 51%, restoring sensory transduction and morphology in inner hair cells, with a transient rescue of low-frequency hearing observed 4 weeks post-injection	Yeh et al. (2020)
HL	Myo6^C442Y/+^ mouse	mxABE	Inner ear injection of AAV-PHP.eB	Increased survival rate of inner ear hair cells, improved mouse hearing, and therapeutic effect lasted for up to 3 months	Xiao et al. (2022)
β-Thalassemia	Patient-derived CD34^+^ cells	hA3A-BE3	Electroporation RNP	Following differentiation of patient-derived CD34 cells, the γ-globin level increased from ∼6.8% to ∼ 44.2%	Wang et al. (2020)
β-Thalassemia and SCD	Patient-derived CD34+HSPCs	A3A (N57Q)-BE3	Electroporation RNP	Approximately 90% editing efficiency was achieved in patient-derived CD34^+^ HSPCs, with multiplex editing at the BCL11A erythroid enhancer and the HBB -28A>G promoter	Zeng et al. (2020)
β-Thalassemia	CD46/β-YAC mouse	AncBE4max and ABEmax	Intravenous injection of the HDAd5/35++ vector	*In vivo*, an average of 20% of target sites in HSPCs were edited, resulting in normal expression of fetal hemoglobin without detectable off-target editing	Li et al. (2021a)
β-Thalassemia and SCD	CD46/β-YAC mouse	HDAd-ABE8e	Intravenous injection of the HDAd5/35++ vector	*In vivo* HSC base editing in CD46/β-YAC mice resulted in >60% −113 A>G conversion, effectively activating the expression of γ-globin	Li et al. (2022a)
SCD	Patient-derived CD34^+^ HSPCs, NBSGW mouse, Townes mouse	ABE8e-NRCH	Electroporation of RNP or mRNA	Editing frequency in HSPCs was 80%, and 16 weeks after transplantation into immunodeficient mice, the frequency remained at 68%. After editing the HSPCs of humanized SCD mice, a secondary transplantation still showed long-term efficacy	Newby et al. (2021)
SCD	HUDEP2^∆εγδβ^ cell line,	ABE7.10 and ABE8e	Electroporation RNP	−175A > G base editing results in HbF reaching 80%–90%	Mayuranathan et al. (2023)
β-Thalassemia	Patient-derived CD34^+^ HSPCs	ABE8e and ABE8e-SpRY	Electroporation RNP	Editing efficiencies before transplantation for ABE8e and ABE8e-SpRY were 90.7% and 77.6%, respectively. After 16 weeks post-transplantation, the proportion of edited β-hemoglobin recovered to 66.7% and 76.3%	Liao et al. (2023)
PKU	Pah^enu2^ mouse	SaKKH-BE3	Intravenous injection of dual AAV8	Blood phenylalanine levels below 120 μmol/L	Villiger et al. (2018)
PKU	Pah^enu2^ mouse	SaKKH-BE3	Intravenous injection of dual AAV2/8 or LNP	Verification that SaKKH-CBE3 did not cause significant off-target RNA and DNA mutations, and no malignant transformation of the liver was observed	Villiger et al. (2021)
FH	C57BL/6J mouse	BE3	Retro-orbital injection of Ad	Base editing reached up to 34%; within 4 weeks, PCSK9 levels decreased by 54%, and cholesterol decreased by 28%	Chadwick et al. (2017)
FH	hPCSK9-KI mouse	BE3	Intravenous injection of Ad	Editing frequency at the PCSK9 site ranged from 11.1% to 34.9%	Carreras et al. (2019)
FH	Cynomolgus monkeys	ABE8.8	Intravenous injection of LNP	In cynomolgus monkeys, levels of PCSK9 and low-density lipoprotein cholesterol in the blood decreased by approximately 90% and 60% respectively, and remained stable for at least 8 months	Musunuru et al. (2021)
FH	C57BL/6J mouse Cynomolgus monkeys	ABEmax	Intravenous injection of LNP	Plasma PCSK9 and LDL levels were stably reduced by 95% and 58% in mice and by 32% and 14% in macaques	Rothgangl et al. (2021)
DCM	RBM20^R634Q^ iPSCs, RBM20^R634Q^ mouse	ABEmax-VRQR-SpCas9	Intraperitoneal injection of AAV9	In iPSCs, the editing efficiency of RBM20 reaches up to 92%; *In vivo* editing results in precise correction of 66% of RBM20 cDNA transcripts	Nishiyama et al. (2022)
CD3δ SCID	HSPCs(*CD3D* c.202C>T)	ABEmax-NRTH	Electroporation	HSPCs editing level was (71.2% ± 7.85%). After 16 weeks post-transplantation, the editing frequencies in the bone marrow, spleen, and thymus were 84.5% ± 5.52%, 78.2% ± 6.18%, and 87% ± 13.1%, respectively	McAuley et al. (2023)
SMA	Δ7SMA mouse	ABE8e-SpyMac	Intracerebroventricular injection of dual AAV9	Induced 87% of T6>C conversions, improving motor function, and extending average lifespan by approximately 33%	Arbab et al. (2023)
SMA	SMNΔ7 mouse	ABE8e-SpRY	Intracerebroventricular injection of AAV9 or AAV-F^60^	*In vivo* editing includes the brain, spinal cord, liver, heart, and skeletal muscles, with approximately 4% in the spinal cord and approximately 6% in the brain	Alves et al. (2023)
SMA	SC-SMA^T5C^ mouse	TadA-TadA*-SaCas9n-KKH	Microinjection	Compared to unedited SMA mice (which typically die within 14 days), SC-SMA^T5C^ mice showed an extended lifespan of approximately 400 days	Lin et al. (2020)

### 5.1 Duchenne muscular dystrophy

Duchenne muscular dystrophy (DMD) is a progressive genetic muscle disorder caused by mutations in the gene encoding the dystrophin protein on the X chromosome (Long et al., 2018; Verhaart and Aartsma-Rus, 2019). The *DMD* gene is the largest known human gene, spanning 2.4 megabases, and its extensive size increases the likelihood of mutations, with the majority of *DMD* mutations being due to deletions or duplications (Bladen et al., 2015). Dystrophin, the protein encoded by this gene, is essential for stabilizing and protecting muscle cells. Mutations in the dystrophin gene lead to the production of non-functional dystrophin, compromising the structural integrity of muscle cell membranes and resulting in the progressive destruction of muscle cells (Verhaart and Aartsma-Rus, 2019). As one of the most common lethal genetic disorders, approximately one in every 3,500 to 5,000 newborn males are affected by DMD (Mendell et al., 2012). Despite extensive research, both basic and clinical, an effective and precise treatment for DMD remains elusive. Corticosteroids (such as prednisone or deflazacort) are one of the main methods of treatment for DMD, widely used to delay the loss of muscle function (Roberts et al., 2023). They primarily provide direct and indirect protection to muscle cells by inhibiting inflammatory responses and through immunomodulatory effects (Ricotti et al., 2013; McDonald et al., 2018). However, long-term use of corticosteroids may lead to a range of side effects, including weight gain, osteoporosis, and insulin resistance (Ricotti et al., 2013). Eteplirsen and Golodirsen are two exon-skipping agents approved by the United States Food and Drug Administration (FDA), which work by encouraging cells to skip over mutated exons during the mRNA splicing process, providing a treatment option for DMD patients with specific exon mutations (Nelson and Miceli, 2017; Heo, 2020; Shirley, 2021). Although exon-skipping therapy offers a new treatment approach, its applicability is limited, and the development and production costs are high. CRISRPR-mediated exon knockout restores expression and function of dystrophin at the cellular and animal levels (Xu et al., 2016; El Refaey et al., 2017), but Cas9-induced double-strand breaks may lead to large genomic deletions and even chromosomal rearrangements (Shin et al., 2017; Kosicki et al., 2018). As our comprehension of the underlying mechanisms of DMD advances and gene editing technology matures, the application of base editing in DMD treatment is emerging as a focal area of research, representing a promising frontier in the quest for an effective therapy.

The base editor has demonstrated efficient and precise repair of duchenne muscular dystrophy gene mutations in mouse models. This achievement led to the restoration of dystrophin protein expression and subsequent improvement of symptoms in the mice, showcasing the potential of this technology as a therapeutic approach for DMD. In a study by Ryu and colleagues, *in vivo* base editing was used to correct a DMD-causing nonsense mutation in exon 20 of the *Dmd* gene in mice (Ryu et al., 2018). They employed extended gRNAs to expand the editing window of ABE7.10. When ABE7.10 was delivered into the myoblasts of a DMD mouse model using a double trans-splicing adeno-associated virus (tsAAV) (Sun et al., 2000; Lai et al., 2005) vector system, it restored dystrophin protein expression in approximately 17% ± 1% of myofibers (Ryu et al., 2018). This level of restoration is particularly significant, as achieving more than 4% of normal dystrophin protein expression is considered sufficient to improve muscle function in DMD mice (van Putten et al., 2013). Xu et al. then tested the feasibility and long-term efficacy of systemic iABE-NGA-based therapy in the DMD mouse (mdx^4cv^) model (Xu L. et al., 2021). They packaged iABE-NGA into a double AAV9 vector mediated by the intronic peptide Gp41-1 (referred to as AAV9-iNG) and administered it via tail vein injection. Remarkably, 10 months post-injection, over 95% of cardiac myocytes in mdx^4cv^ mice showed restored dystrophin expression, with about 15% dystrophin reconstitution also observed in skeletal muscles (Xu L. et al., 2021). These mice exhibited reduced myocardial fibrosis and enhanced muscle contractile function, significantly ameliorating the pathological characteristics of their muscle tissue. Additionally, the study assessed potential immune responses and off-target effects in the mdx^4cv^ mice treated with AAV9-iNG. The results indicated no significant toxic side effects or notable genomic off-target events, underscoring the safety and precision of this therapeutic approach.

**FIGURE 6:**
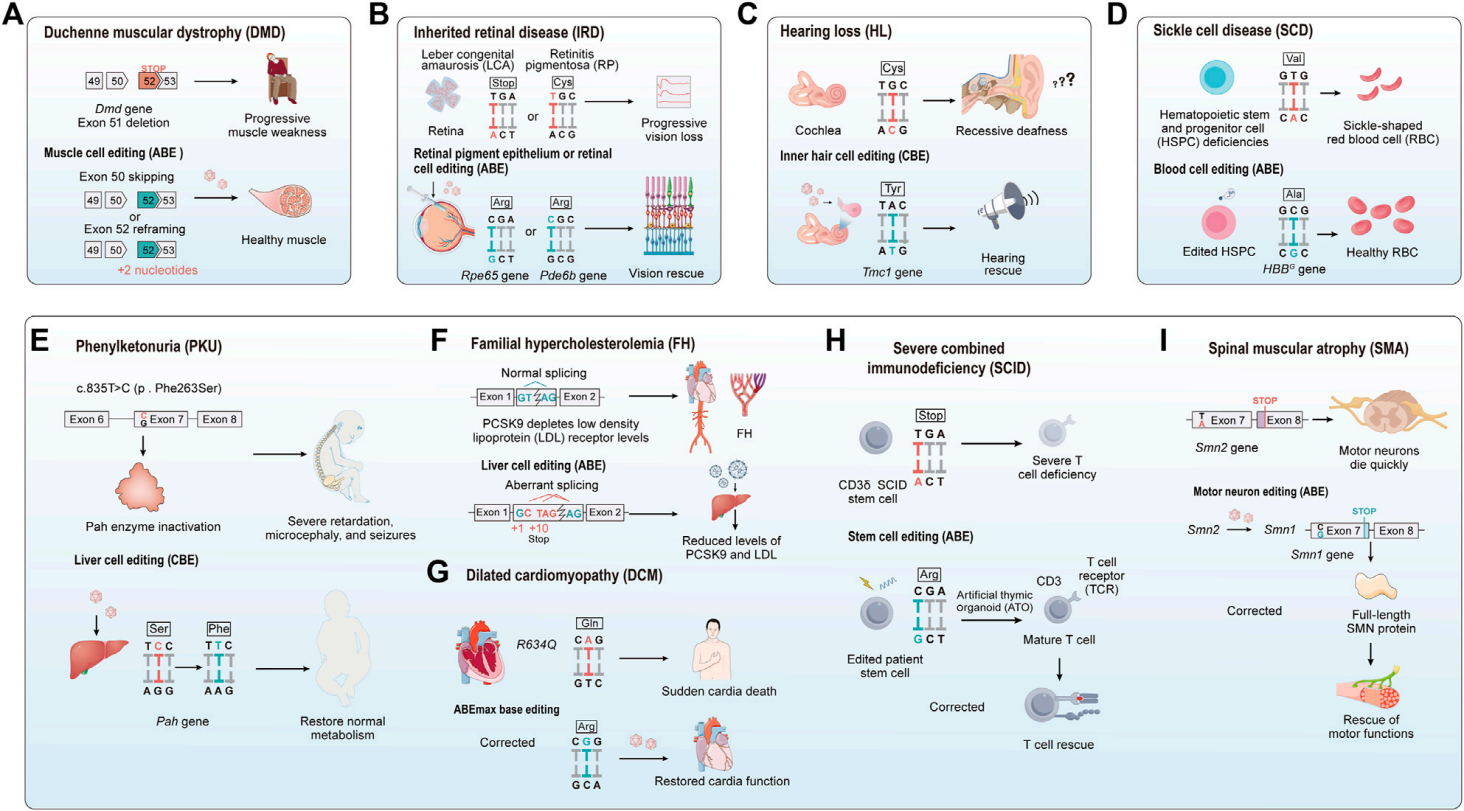
The application of base editing in treating genetic diseases. **(A)** Duchenne muscular dystrophy (DMD): The absence of axon 51 leads to a premature stop codon in exec 52. Base editing induces exec slapping at either non 50 or 52 to construct a correct open reading frame, thereby improving or restoring muscle function. **(B)** Inherited retinal disease (IRD): For IRD, base editors are employed to precisely target and cared specific mutations vnthin retinal genes. The goal is to restore normal retinal function or to halt further degeneration of the retina. **(C)** Genetic heanng loss (HL): The application of base editing technology in correcting gene detects causing hearing loss, with the goal of restoring or preserving auditory function. **(D)** Sickle cell disease (SCD): Base editing modifies the pathogenic protein into benign variants like HbS to HbG-Makassar, to treat red blood cell disorders. **(E–I)** For phenyketonuria (PKU), familial hypercholesterolemia (FH), dilated cardiomyopathy (DCM), CD3δ severe combined immunodeficiency (CD3δ SCID), and spinal muscular atrophy (SMA): The figure illustrates the utilization of base editing in targeting specific mutations associated with these diseases. The applications are diverse, ranging from restoring liver metabolic functions, reducing cholesterol levels, preventing cardiovascular diseases, to preserving cardiac, immune, and muscle functions.

In addition to correcting point mutations in the *Dmd* gene, base editors have the ability to restore dystrophin expression in DMD by inducing exon skipping. Gapinske and colleagues developed a base editing approach named CRISPR-SKIP, which achieves permanent exon skipping by introducing C to T or A to G mutations at splice acceptor sites in genomic DNA (Gapinske et al., 2018). Based on this strategy, Gapinske and colleagues implemented a split-intein mediated dual AAV system to deliver the ABE and CBE base editors, successfully achieving the editing of exon 45 in the *DMD* gene both in human cells and *in vivo* in mice (Gapinske et al., 2023). In another study, the team led by Chemello utilized a dual AAV9 delivery system to administer ABEmax-SpCas9-NG to a DMD mouse model with exon 51 deletion, targeting the *Dmd* gene for exon skipping (exon 49 spliced to exon 52), resulting in the restoration of dystrophin expression in 96.5% ± 0.7% of muscle fibers (Figure 6A) (Chemello et al., 2021). These innovative therapeutic strategies expand the range of genome editing techniques available for treating DMD.

Historically, respiratory failure was the leading cause of death in DMD patients, but with advancements in respiratory support, cardiac failure has now emerged as the primary cause of morbidity and mortality (Eagle et al., 2002; McNally et al., 2015). Currently, cardiac symptoms in DMD patients still lack effective therapeutic interventions. Yuan et al. utilized targeted AID-mediated mutagenesis (TAM) to achieve exon 50 skipping in induced pluripotent stem cells (iPSCs) derived from DMD patients (Yuan et al., 2018). This approach restored dystrophin protein expression and function in iPSC-derived cardiomyocytes. They also identified a Dmd^E4*^ mouse model, which mimics the cardiac pathology of DMD, exhibiting progressive cardiac dysfunction similar to that in human patients (Li J. et al., 2021). Intraperitoneal injection of the AAV9-packaged base editor eTAM into postnatal day 2 or day 3 Dmd^E4*^ mice achieved skipping of *Dmd* exon 4 (exon skipping efficiency of 59.98% ± 4.74%), which modestly restored dystrophin protein expression. Dystrophin levels in the hearts of Dmd^E4*^ mice remained close to wild-type levels after 12 months, alleviating the dystrophic condition and prolonging survival (Li J. et al., 2021). Wang et al. created a DMD hiPSC cell line with exon 48 to 54 deletions (ΔE48-54) using CRISPR-Cas9 (Wang P. et al., 2023). They restored dystrophin expression in DMD hiPSC-derived cardiomyocytes to 42.5% ± 11% of wild-type levels by inducing exon 55 skipping with the ABE8eV106W-SpCas9 base editor (Wang P. et al., 2023). Furthermore, Gapinske and colleagues accomplished systemic delivery of ABE8e-UGI using the MyoAAV system with RGD motifs, achieving DNA editing in cardiac tissue (Gapinske et al., 2023).

RNA editing also shows great promise in DMD treatment. In a study conducted by Li and colleagues, a previously uncharacterized c.4174C>T nonsense point mutation in the *DMD* gene was identified and its pathogenicity was confirmed in the humanized DMD^E30mut^ mouse model (Li et al., 2023). Utilizing a mini-dCas13X-mediated RNA adenine base editing system (mxABE), they achieved up to 84% A-to-G editing efficiency in the DMD^E30mut^ mice, leading to differential restoration of dystrophin protein expression in various muscle tissues including the diaphragm, tibialis anterior, and heart (Li et al., 2023). Continuous treatment with mxABE can restore the expression of dystrophin protein to 50% of wild-type levels in DMD^E30mut^ mice, significantly improving muscle growth and function in the mice. This study strongly suggests that mxABE-based strategies can be used to effectively treat genetic diseases caused by nonsense point mutations. In a similar vein, another small compact RNA base editor (ceRBE) successfully repaired the Q1392X mutation in the DMD gene in DMD^Q1392X^ humanized mice (68.3% ± 10.1%), in which dystrophy were rescued to a high level of 68.1% ± 9.4% in right tibialis anterior tissue (Wang et al., 2023c). These studies underscore the potential of RNA editing, particularly mxABE and ceRBE systems, as powerful tools for treating DMD and possibly other genetic diseases.

### 5.2 Inherited retinal diseases

Inherited retinal diseases (IRDs) are a genetically and clinically heterogeneous group of disorders that affect the function and structure of the retina, leading to visual impairment and even blindness (Sahel et al., 2014). Owing to the heterogeneity of IRDs, their clinical manifestations exhibit considerable variability, encompassing diverse symptoms, severity levels, and modes of inheritance (Pulman et al., 2022; Jo et al., 2023a). Examples of these disorders include Leber congenital amaurosis (LCA), retinitis pigmentosa (RP), color blindness, and choroideremia, among others. The identification of hundreds of genes associated with IRDs underscores the genetic complexity of these conditions, complicating both diagnosis and treatment. Traditional approaches to managing IRDs, such as visual aids, gene therapy, and cell transplantation, are constrained in their effectiveness and accompanied by numerous challenges (Yue et al., 2016; Gasparini et al., 2019; Singh et al., 2021).

LCA is a rare, severe genetic retinal disorder, often considered the earliest and most severe form within IRDs. It primarily exhibits autosomal recessive inheritance and is characterized by progressive retinal degeneration and vision loss (Cremers et al., 2002). LCA is caused by critical gene mutations responsible for the development and function of the retina or retinal pigment epithelium (RPE), with most LCA patients experiencing severe visual impairments in infancy or early childhood, leading to complete blindness usually by the age of 30–40 (den Hollander et al., 2008). RPE-specific 65-kDa protein (RPE65) is an essential isomerase in the classical visual cycle, playing a pivotal role in converting 11-cis-retinol to 11-cis-retinal (Redmond et al., 1998). Loss-of-function mutations in the *RPE65* gene disrupt this crucial process, impairing the visual cycle and leading to a range of retinal diseases (Redmond et al., 1998). Among these, mutations in *RPE65* are a significant cause of leber congenital amaurosis type 2 (LCA2), accounting for about 16% of all LCA cases (Choi et al., 2022). Luxturna (voretigene neparvovec-rzyl) is an FDA-approved treatment for LCA and other retinal diseases caused by mutations in the RPE65 gene (FDA, 2017b). It aims to deliver a normal RPE65 gene directly into the eye in a one-time procedure, compensating for the gene that has lost function due to mutation. It is worth noting that clinical trials for Luxturna included incidents of mild ocular discomfort, and the FDA’s prescribing information for Luxturna contains specific warnings about serious ocular adverse events (FDA, 2017c; Russell et al., 2017). The efficiency of *Rpe65* nonsense mutation correction mediated by CRISPR-Cas9 is very low and is associated with a high incidence of indels (Jo et al., 2019). Therefore, there is a broader therapeutic demand for the treatment of LCA. Research has demonstrated that base editing can effectively repair mutations in the *Rpe65* gene, thereby improving visual function in mouse models of LCA (Suh et al., 2021; Choi et al., 2022; Jo et al., 2023b). Suh and colleagues used rd12 mice, an LCA model with a nonsense mutation in *Rpe65* gene (c.130C>T; p. R44X), to demonstrate the efficacy of base editing (Figure 6B) (Suh et al., 2021). Utilizing a LV vector, they delivered the base editor ABEmax subretinally, achieving a 29% correction of the mutant gene in this model with no observable off-target effects. Subsequent assessment revealed the reinstatement of the visual cycle, preservation of the visual pathway from the retina to the primary visual cortex, and recovery in cortical responses in treated rd12 mice. Jo et al. employed a dual AAV system-mediated base editor, NG-ABE, in rd12 mice (Jo et al., 2023b). This approach effectively corrected *Rpe65* mRNA transcripts (80%) and restored visual function, with retinal electroretinogram (ERG) wave amplitudes reaching 60% of those wild-type mice. While both ABEmax and NG-ABE strategies successfully improved visual function in rd12 mice, it remains unclear whether they can prevent further degeneration of retinal photoreceptors. In another study, an optimized NG-ABE base editor was delivered via LV vector in rd12 mice, successfully correcting the *Rpe65* mutation and leading to the re-expression of the truncated Rpe65 protein, thereby restoring cone cell function (Choi et al., 2022). These findings suggest that base editing can correct pathogenic mutations and potentially offer long-term protection to the retina, preventing photoreceptor degeneration. The exploration of nonviral vectors for delivering base editors in the treatment of LCA marks a significant step towards enhancing safety and specificity. An illustrative example is the use of the purified NG-ABEmax RNP complex, which has been applied to the rd12 mouse retina for gene correction (Jang et al., 2021). This approach offers a notable advantage by avoiding the accidental, random integration of DNA and reducing cellular immune responses caused by excess sgRNA, thereby improving the safety profile of base editing. Further advancing this nonviral delivery approach, Kabra and colleagues utilized silicon nanocapsules (SNCs) to deliver the ABE8e base editor (Kabra et al., 2023). They focused on editing the W53X mutation in the *Kcnj13* gene of LCA16 mice (*Kcnj13* ^*W53X/+ΔR*^), which led to the restoration of Kir7.1 potassium ion channel function in the RPE. This intervention not only improved the functionality of the RPE in diseased mice but also underscored the potential of nonviral delivery systems in preserving vision in LCA16. This study and others like it represent important advancements in the field of gene therapy, offering safer and more specific alternatives to traditional viral vector-based methods.

RP is a common genetically heterogeneous retinal disorder, characterized by the progressive loss of rod and cone photoreceptor cells (Hartong et al., 2006). Initially manifesting as night blindness, RP gradually progresses to a more severe loss of vision, ultimately resulting in total blindness (Hartong et al., 2006). To date, over 100 genes have been identified in various subtypes of RP with different genetic patterns, including the rhodopsin gene (*RHO*), the pre-mRNA processing factor 31 gene (*PRPF31*), and the peripherin 2 gene (*PRPH2*), among others (Bhardwaj et al., 2022; Qin et al., 2023b). Although Luxturna offers a treatment option for a minority of RP patients carrying *RPE65* mutations, the genetic diversity of RP means that a universal cure for all patients is currently lacking (Cross et al., 2022). Against this backdrop, base editing technology provides new hope for correcting pathogenic genes, demonstrating potential for the treatment of RP. Mutations in the *PDE6B* gene impact the protein that is the β-subunit of the rod cell cyclic guanosine monophosphate (cGMP)-phosphodiesterase (PDEβ), with such mutations being one of the common causes of autosomal recessive inherited RP (Danciger et al., 1995). The rd10 mouse is a commonly used model for RP, characterized by a missense mutation (c.1678C>T, p. R560C) in its *Pde6b* gene, exhibiting a phenotype similar to that of typical human RP patients (Chang et al., 2007; Gargini et al., 2007). Su and colleagues employed the retinal tropism vector AAV8 for subretinal delivery of NG-ABE8e in rd10 mice, achieving an effective correction of the pathogenic mutation in the *Pde6b* with a correction efficiency of up to 54.97% at the cDNA level (Figure 6B) (Su et al., 2023). The application of NG-ABE8e not only corrects the mutation in the *Pde6b* gene in rd10 mice but also preserves both rod and cone cells, significantly improving the retinal structure and visual behavior in the treated mice. In another related study, Wu and colleagues utilized a dual AAV5 vector system to precisely correct the *Pde6b* mutation in rd10 mice through the application of SpRY-ABE8e (Wu et al., 2023). They achieved an average cDNA editing efficiency of 34.07% ± 7.12%, resulting in the restoration of functional protein expression, extended survival of photoreceptor cells, and enhanced visual function.

Aniridia is a rare congenital eye disorder predominantly associated with dominant loss-of-function mutations in the transcription factor paired box 6 (PAX6) (Hingorani et al., 2012). It is characterized by the absence or underdevelopment of the iris and may also exhibit other ocular abnormalities, including cataracts, retinal pigmentary degeneration, and other eye-related anomalies (Hingorani et al., 2012). Previous studies have indicated that the use of nonsense suppression and mitogen-activated protein kinase kinase (MEK or MAP2K) inhibitors can rescue the retinal and visual function in a mouse model of aniridia (Wang X. et al., 2017; Cole et al., 2022). More recently, Adair and colleagues developed a humanized aniridia mouse model (CHuMMMS) and a corresponding cell line (Adair et al., 2023). In the study conducted, researchers successfully achieved a high genome correction rate, averaging 76.8% ± 0.48%, by employing the adenine base editor ABE8e (Adair et al., 2023). Additionally, the study involved the use of LNP-mediated ABE8e to restore Pax6 protein expression in primary neurons, attaining a correction rate of 24.8%.

### 5.3 Genetic hearing loss

Genetic hearing loss (HL) is an auditory impairment resulting from genetic mutations or abnormalities, primarily classified into syndromic hearing loss (30%) and non-syndromic hearing loss (70%) based on inheritance patterns and clinical manifestations (Yang et al., 2019). Syndromic hearing loss involves auditory impairment along with pathological changes in other systems or organs, whereas non-syndromic hearing loss manifests solely as auditory impairment (Tropitzsch et al., 2023). Traditional treatments for genetic HL, such as sound amplification devices or cochlear implants that stimulate the auditory nerve, provide limited improvement. They improve hearing but do not restore the natural functionality of the ear. In terms of genetic interventions, CRISPR/Cas9-mediated HDR and NHEJ have shown potential in silencing or knocking out dominant pathogenic mutations, offering benefits in genetic HL treatment (Mianné et al., 2016; Noh et al., 2022; Xue et al., 2022). However, these methods generally do not address mutations that cause recessive functional loss. Recessive genetic HL requires correction rather than disruption or silencing of pathogenic alleles to prevent hearing loss, posing a series of challenges in the treatment of genetic HL. Mutations in the transmembrane channel-like 1 (*TMC1*) gene can result in dominant or recessive deafness, as the encoded protein is closely linked to the formation of mechanosensitive ion channels involved in auditory sensation (Corey et al., 2019). Recessive mutations in the *TMC1* gene lead to rapid degeneration of hair cells, resulting in swift and complete deafness, with the *TMC1* mutations accounting for 4% to 8% of hereditary deafness in certain populations (Kitajiri et al., 2007; Sirmaci et al., 2009). Treating mice with recessive *Tmc1* mutations via AAV gene therapy partially restores hearing, but the effect is limited in duration and does not genuinely edit or repair recessive gene mutations (Askew et al., 2015; Landegger et al., 2017). David Liu and colleagues achieved a significant breakthrough by successfully repairing *Tmc1* gene mutations in the inner ear of Baringo mice through the application of base editing technology (Figure 6C) (Yeh et al., 2020). This achievement represents the first successful application of a base editor in treating recessive hearing loss. The Baringo mice (*Tmc1* ^*Y182C/Y182C*^ *; Tmc2* ^*+/+*^) harbor a TA to CG mutation in the *Tmc1* gene, resulting in the onset of severe deafness by 4 weeks of age (Manji et al., 2012). The team utilized a dual AAV strategy to deliver the optimized adenine base editor, AID-BE4max, featuring enhanced AID deaminase, into the inner ear of Baringo mice, successfully achieving a notable 50% editing efficiency in the *Tmc1* sequence (Yeh et al., 2020). The AID-BE4max treatment restored inner hair cell sensory transduction and cell morphology in the mice, temporarily rescuing low-frequency hearing. However, the therapeutic effect lasted only for about 4 weeks, underscoring the need for further enhancements in efficiency and durability for this strategy to advance as a viable treatment for hereditary deafness.

The *MYO6* gene encodes an atypical myosin, Myosin IV, serving as a molecular motor expressed in inner ear hair cells and playing a crucial role in auditory and vestibular function (Ahmed et al., 2003). Pathogenic variations in the *MYO6* gene have been found to be associated with autosomal dominant or recessive hereditary hearing loss (Melchionda et al., 2001; Ahmed et al., 2003). In a significant study, Xiao et al. utilized an AAV-mediated mxABE to precisely correct the *Myo6* gene mutation in the inner ear of Myo6^C442Y/+^mice, a model for autosomal dominant hereditary deafness (Xiao et al., 2022). The mxABE editing technique enhanced the survival rate of inner ear hair cells and ameliorated the auditory function in mice, with the therapeutic effects persisting for a duration of up to 3 months. This therapeutic approach underscores the substantial potential of RNA base editing as a treatment for dominant hereditary hearing loss, further supporting the development and application of RNA correction therapies in genetic medicine.

### 5.4 Hereditary blood disorders

Hematological hereditary diseases, originating from or impacting the hematopoietic system and accompanied by hematological abnormalities, are characterized by anemia, hemorrhage, fever, and coagulation disorders. Among these, SCD and β-thalassemia are notably common. SCD is caused by a specific mutation in the β-globin gene (*HBB*), leading to the production of abnormal hemoglobin (HbS), with symptoms including, but not limited to, pain crises, anemia, increased risk of infections, and organ damage (Bauer and Orkin, 2015). β-thalassemia, similarly resulting from mutations in the *HBB* gene, impairs the normal synthesis of β-globin, leading to its deficiency and causing symptoms such as chronic anemia, fatigue, growth delay, and facial bone deformities (Saraf et al., 2014). The persistent presence of fetal hemoglobin (HbF), comprised of γ-globin and α-globin, is closely correlated with the severity of SCD and β-thalassemia (Xu et al., 2011; Bauer et al., 2013; Canver et al., 2015). Elevating the levels of HbF within patients is one of the key strategies for the treatment or mitigation of these types of anemia. The γ-globin gene, encoded by the *HBG* gene and typically silenced in adulthood, is functional in fetal stages and can compensate for β-globin deficiency (Fontana et al., 2023). The *BCL11A* gene plays a crucial role in repressing γ-globin gene expression in erythrocytes (Bauer et al., 2013). Consequently, therapeutic strategies that involve reactivating γ-globin gene expression in patients to supplement insufficient β-globin and employing targeted editing of the *BCL11A* gene to reactivate HbF present viable methods for addressing these disorders. Hydroxyurea, one of the first-line treatment options approved by the FDA for SCD, effectively reduces the incidence of pain crises and the need for transfusions by increasing fetal hemoglobin levels (Charache et al., 1995; Steinberg et al., 2003; Lee and Ogu, 2022). Although hydroxyurea demonstrates significant benefits in treating SCD, its use is also accompanied by some drawbacks and potential side effects, such as dose dependency, bone marrow suppression, and reproductive toxicity (Brawley et al., 2008). L-glutamine, another medication approved by the FDA for the treatment of SCD following hydroxyurea, offers patients a new path to relief by reducing oxidative stress (FDA, 2017a; Niihara et al., 2018). However, its side effects include conditions such as nausea, diarrhea, and abdominal pain (Quinn, 2018; Ogu et al., 2021). In recent years, the FDA and the European Medicines Agency (EMA) have approved two innovative treatments for SCD: the monoclonal antibody Crizanlizumab and the small molecule compound Voxelotor (FDA, 2019; EMA, 2020a). The former reduces the incidence of pain crises by inhibiting the adhesion of red blood cells to the vascular wall, while the latter alleviates the sickling of red blood cells by increasing their affinity for oxygen (Oksenberg et al., 2016; Ataga et al., 2017; Kutlar et al., 2019; Vichinsky et al., 2019). However, both Crizanlizumab and Voxelotor may cause side effects such as headaches, fever, and chest pain (Ataga et al., 2017; Vichinsky et al., 2019). For β-thalassemia, traditional treatment strategies primarily rely on periodic blood transfusions, which necessitate chelation therapy to manage the consequent iron load accumulation and prevent organ damage from iron overload (Cappellini et al., 2018). While these treatments are effective in the short term, they do not cure the disease and require lifelong management. Luspatercept, an innovative recombinant fusion protein that promotes the maturation of late-stage red blood cells and increases hemoglobin levels, offers a new treatment avenue for transfusion-dependent β-thalassemia (Markham, 2020; Sheth et al., 2023). Although it opens new paths for the treatment of transfusion-dependent β-thalassemia, it may lead to adverse events such as bone pain, joint pain, and hyperuricemia (Cappellini et al., 2020). Furthermore, hematopoietic stem cell transplantation offers a potential curative treatment for SCD and β-thalassemia under certain conditions, but its high risk and limited applicability restrict its widespread use (Chou, 2013; Cappellini et al., 2018).

Base editing technology facilitates precise modification of the BCL11A erythroid enhancer, attenuating its regulatory function and elevating HbF expression levels. The transcription factor GATA1 is involved in regulating red blood cell development and hemoglobin synthesis (Crispino and Horwitz, 2017). Mutations in the GATA1 binding sites on the erythroid-specific enhancer of BCL11A result in diminished regulatory activity on the *BCL11A* gene (Canver et al., 2015). David Liu and colleagues achieved successful introduction of two point mutations in a GATA1 binding site at the +58 BCL11A erythroid enhancer by employing the adenine base editor ABE8e (Richter et al., 2020). This resulted in simultaneous editing of two target adenines in 54.4% ± 12.5% of the alleles. In a related study, Zeng et al. employed electroporation to introduce the A3A(N57Q)-BE3 base editor into CD34^+^ hematopoietic stem/progenitor cells (CD34^+^ HSPCs) from patients with SCD and β-thalassemia, targeting and editing the +58 BCL11A erythroid enhancer (Zeng et al., 2020). This study led to the downregulation of BCL11A expression in red blood cells, effectively inducing the expression of γ-globin and HbF. Furthermore, the researchers conducted dual-site editing at −28 HBB and +58 BCL11A in β-thalassemia HSPCs, leading to the *in vivo* differentiation of cells producing functionally normal hemoglobin (Zeng et al., 2020).

Base editing has shown promise in introducing hereditary persistence of fetal hemoglobin (HPFH) mutations into the HBG1/2 promoter region, effectively increasing the expression of HbF. HPFH is typically caused by deletions in the β-globin gene cluster and point mutations in the γ-globin gene promoters, characterized by the persistent presence of excess HbF in adult red blood cells (Forget, 1998). Patients with HPFH generally do not exhibit severe anemia symptoms, and in some cases, the persistence of HbF can even provide a protective effect (Lu et al., 2022). Wang et al. delivered the base editor hA3A-BE3 to healthy or patient-derived CD34^+^ HSPCs via electroporation, targeting the HBG1/2 promoters to induce HPFH (Wang et al., 2020). This editing led to a substantial elevation in γ-globin levels in patient-derived cells, increasing from 6.8% to 44.2%, reaching a level that has potential clinical benefits for patients with β-hemoglobinopathies. Li and colleagues advanced the application of base editing for *in vivo* induction of HPFH mutations, promoting β-globin production (Li C. et al., 2021). They initially used the HDAd5/35++ vector to deliver base editors AncBE4max and ABEmax, targeting the +58 BCL11A erythroid enhancer or reconstructing HPFH mutations in the HBG1/2 promoters. This resulted in reactivated γ-globin in HUDEP-2 erythroid progenitor cells. Following this, β-YAC mice (Peterson et al., 1993) were treated using HDAd-ABE-sgHBG-2, achieving an average editing efficiency of 20% in HSPCs and leading to normal fetal hemoglobin expression with no detectable off-target effects (Li C. et al., 2021). In another study conducted by Li and colleagues, CD34 cells derived from β-thalassemia and SCD patients were transduced with an HDAd5/35++ vector expressing ABE8e, successfully introducing the −113 A>G HPFH mutation into these cells (Li C. et al., 2022). In β-YAC/CD46 mice, a single intravenous injection of the HDAd-EF1α.ABE8e vector achieved an *in vivo* hematopoietic stem cell (HSC) editing efficiency of up to 60%, with 30% γ-globin of β-globin expressed in 70% of erythrocytes (Li C. et al., 2022). Furthermore, utilizing base editors to create new transcription factor binding sites in the γ-globin promoter represents another viable strategy for inducing HbF expression (Cheng L. et al., 2021; Ravi et al., 2022).

Base editing offers another promising strategy for treating hemoglobinopathies, like SCD, by converting pathogenic hemoglobin variants into benign forms. This approach involves transforming the aberrant SCD β-globin gene (*HBB* ^*S*^) into a naturally occurring, non-pathogenic variant, such as the Makassar β-globin gene (*HBB* ^*G*^) (Viprakasit et al., 2002; Chu et al., 2021; Newby et al., 2021). In a study by Chu and colleagues, the research team employed deaminase-inlaid base editors (IBEs) on fibroblasts derived from a patient with homozygous SCD (Chu et al., 2021). This process achieved an impressive Makassar editing efficiency of up to 50%. Furthering this approach, Newby and his team employed ABE8e-NRCH mRNA and sgRNA through electroporation into CD34^+^ HSPCs obtained from SCD patients (Figure 6D) (Newby et al., 2021). This intervention successfully converted 80% of *HBB* ^*S*^ to *HBB* ^*G*^. Following the transplantation of these base-edited hematopoietic stem cells into SCD mouse models, the frequency of HBB^G^ editing was maintained at 68% even after 16 weeks, effectively preserving the hematopoietic stem cells (Newby et al., 2021). The study also performed secondary transplantation of these edited stem cells, demonstrating that the modified cells retained functionality comparable to healthy hematopoietic stem cells. This finding not only underscores the efficacy of base editing in treating SCD but also highlights the durability and stability of the edited cells, marking a significant advancement in gene therapy for blood disorders.

To optimize genome editing for HbF, David Liu and colleagues compared five different gene editing approaches that were mediated either by Cas9 nucleases or adenine base editors (Mayuranathan et al., 2023). Their findings revealed that the adenine base editor of γ-globin-175A > G was the most effective in inducing HbF. This was further validated by the successful application of this editing approach in regenerated human hematopoietic stem cells within transplanted mice. In these models, the −175A > G editing notably reduced the formation of hypoxia-induced sickle-shaped cells, which are characteristic of SCD. This outcome demonstrates the potential of base editing in directly addressing the underlying cause of SCD. An important aspect of this study was the direct comparison between Cas9 editing and base editing techniques. The team observed that, compared to Cas9 editing, base editing resulted in precise nucleotide changes, exhibiting uniform HbF induction and being independent of TP53-mediated DNA damage response (Mayuranathan et al., 2023). This independence is a significant advantage, as it could reduce potential complications related to DNA repair pathways, which are a concern with traditional Cas9-mediated editing. Therefore, the findings from Mayuranathan and colleagues suggest that base editing could present a superior alternative to Cas9 for therapeutically inducing HbF. This holds great promise for developing more effective and safer treatments for diseases like SCD. As research in this area progresses, base editing technologies may offer new avenues for treating a range of genetic disorders.

### 5.5 Phenylketonuria

Phenylketonuria (PKU) is a genetic disorder caused by a deficiency or reduced activity of phenylalanine hydroxylase (PAH) in the liver, typically inherited in an autosomal recessive manner (van Spronsen et al., 2021). PKU usually exhibits mild or imperceptible symptoms in infancy, but the accumulation of phenylalanine can lead to neurological damage and delayed intellectual development, with severe untreated cases potentially resulting in intellectual disability, behavioral issues, and epilepsy (van Vliet et al., 2019; Klaassen et al., 2021). Currently, the primary treatments for PKU include strict dietary control, pharmacotherapy, enzyme replacement therapy, and gene therapy, which is still under research (Regier et al., 2022). Dietary management is the cornerstone of PKU treatment, aiming to reduce phenylalanine (Phe) levels in the blood by limiting the intake of foods high in phenylalanine. This approach requires patients to adhere to a low-protein diet for life, which, although effective, can significantly impact the quality of life, especially for the growth and development of children (Ashe et al., 2019). Sapropterin dihydrochloride (Kuvan^®^) is a medication approved in the United States and the European Union for the treatment of PKU, working by enhancing the activity of the PAH enzyme to reduce Phe levels in the blood (Burton et al., 2007; FDA, 2014; EMA, 2020b). It is important to note that Kuvan^®^ is only suitable for PKU patients who have a response to tetrahydrobiopterin (BH4) (Levy et al., 2007; Muntau et al., 2019). Palynziq (pegvaliase-pqpz) constitutes an enzyme replacement therapy approved by both the FDA and the EMA (FDA, 2018; Levy et al., 2018; EMA, 2019). The therapeutic efficacy of this treatment is attributed to its principal active ingredient, pegvaliase, a phenylalanine ammonia-lyase (PAL) that has been modified through PEGylation technology. This modification enables the direct conversion of phenylalanine in the bloodstream into safe, metabolizable entities such as trans-cinnamic acid and additional amino acids (Levy et al., 2018). A distinctive feature of this process is its independence from the conventional metabolic pathway of PAH, thus providing an efficacious means of reducing phenylalanine levels in the blood for patients suffering from PAH deficiency. While Palynziq marks a considerable advancement in the therapeutic landscape of PKU, its application is not devoid of risks. The medication is accompanied by a black box warning on its label, explicitly cautioning against the potential for severe allergic reactions, including anaphylactic shock (FDA, 2018; Harding et al., 2018; Thomas et al., 2018). Accordingly, the deployment of this therapeutic strategy necessitates rigorous medical oversight to ensure the safety of patients undergoing treatment and the immediate enactment of appropriate interventions upon the manifestation of any allergic response signs. Gene therapy represents the future direction of PKU treatment, offering a potential cure by repairing or replacing the defective *PAH* gene. Base editors have demonstrated promising capabilities in correcting pathogenic mutations within the livers of PKU model mice (Figure 6E) (Shedlovsky et al., 1993; Villiger et al., 2018; Villiger et al., 2021). Villiger and his research team achieved successful correction of the *Pah* ^*enu2*^ c.835T>C mutation in the PKU mouse model by employing the dual AAV system to deliver the base editor nSaKKH-BE3 (Villiger et al., 2018). Significantly, this intervention effectively maintained blood phenylalanine levels within the physiological range. The study also observed a high mRNA correction rate of up to 63% in mouse liver extracts, reversing disease-associated phenotypes (Villiger et al., 2018). However, concerns about the potential risks associated with cytosine base editing have been raised. Some studies suggest that cytosine base editors might induce RNA mutations in cell lines (Grünewald et al., 2019a; Grünewald et al., 2019b), and the *in vivo* overexpression of the deaminase rAPOBEC1 could have detrimental effects on the mouse liver (Yamanaka et al., 1995; Yamanaka et al., 1996). Addressing these concerns, subsequent investigations focused on evaluating the off-target effects and overall safety of the cytosine base editor SaKKH-CBE3 in the liver (Villiger et al., 2021). Comprehensive RNA sequencing (RNA-Seq) and whole-genome sequencing (WGS) analyses indicated that SaKKH-CBE3 did not cause significant RNA and DNA off-target mutations. Importantly, no evidence of malignant liver transformation was observed. Further expanding the potential therapeutic approaches, researchers explored the delivery of SaKKH-CBE3 via LNPs in Pah^enu2^ mice (Villiger et al., 2021). This method successfully corrected the pathogenic gene, mitigating the pathological phenotype without noticeable off-target effects across the transcriptome and genome. These studies broaden the application scope for cytosine base editors in the treatment of hereditary liver diseases.

### 5.6 Familial hypercholesterolemia

Familial hypercholesterolemia (FH) is a hereditary disease caused by abnormalities in lipoprotein metabolism, primarily characterized by abnormally elevated levels of LDL cholesterol in the blood (Sniderman et al., 2011; Kersten, 2020). LDL is a critical lipoprotein that transports cholesterol and lipids. Excessively elevated LDL levels can cause cholesterol deposition on arterial walls, significantly increasing the risk of atherosclerosis and related cardiovascular and cerebrovascular diseases (Meng et al., 2023). A key factor in this process is proprotein convertase subtilisin/kexin type 9 (PCSK9), predominantly expressed in the liver. When PCSK9 undergoes loss-of-function mutations, it results in lowered levels of LDL cholesterol, thereby reducing the risk of atherosclerotic cardiovascular diseases (Cohen et al., 2005; Meng et al., 2023). In the management of FH, the prevailing therapeutic strategies encompass pharmacotherapy, lifestyle interventions, and, in certain cases, innovative therapeutic approaches (Lan et al., 2023). Pharmacotherapy forms the cornerstone of FH management, with statins serving as the preferred option to lower low-density lipoprotein cholesterol (LDL-C) levels in the blood, including Atorvastatin, Simvastatin, and Rosuvastatin. These medications work by reducing cholesterol production in the liver, effectively lowering LDL-C levels, but they may cause musculoskeletal adverse reactions such as muscle pain and gastrointestinal discomfort (de Sauvage Nolting et al., 2002; Braamskamp et al., 2015; Thompson and Taylor, 2017). As a complement, additional medications like bile acid sequestrants, intestinal cholesterol absorption inhibitors, and PCSK9 inhibitors are available, but these too necessitate long-term use and carry the risk of potential side effects (Huijgen et al., 2010; Nissen et al., 2016a; Nissen et al., 2016b; Watts et al., 2016). On the other hand, dietary improvements, regular exercise, and maintaining a healthy weight can serve as adjunctive therapy methods for FH treatment. However, lifestyle changes alone rarely achieve target LDL-C levels in most FH patients. On the horizon, emergent modalities like gene editing and gene therapy present potential curative prospects by directly addressing the genetic underpinnings of FH through the correction or replacement of the causative gene defects.

In the field of genetic editing, base editing has been successfully applied to introduce nonsense mutations in the *Pcsk9* gene in the liver of mice (Chadwick et al., 2017; Carreras et al., 2019). This intervention led to marked decreases in plasma PCSK9 protein and cholesterol levels, underlining its potential effectiveness in managing cholesterol-related disorders. A notable application of this technology was demonstrated in a non-human primate model, the cynomolgus monkey (Figure 6F) (Musunuru et al., 2021). Researchers achieved pathogenic gene editing in cynomolgus monkeys by intravenously injecting LNPs to deliver the adenine base editor (ABE8.8). This approach led to over 60% editing of the *PCSK9* gene in the liver, resulting in an approximately 90% reduction in blood PCSK9 protein levels. Remarkably, a single dose of this treatment maintained a long-term, stable reduction of LDL cholesterol by about 60% (Musunuru et al., 2021). Furthermore, the delivery of ABEmax using LNP has successfully achieved precise editing of the *PCSK9* gene in liver tissues of mice and cynomolgus monkeys (Rothgangl et al., 2021). This editing resulted in significant reductions in PCSK9 and LDL protein levels in the blood, with decreases of 95% and 58% observed in mice, and 32% and 14% in cynomolgus monkeys, respectively. These results highlight the potential of base editing as a powerful tool in the prevention and treatment of cardiovascular diseases, especially in conditions like Familial Hypercholesterolemia where traditional treatment methods may be limited.

### 5.7 Dilated cardiomyopathy

Dilated cardiomyopathy (DCM) is a severe cardiac disease characterized by an enlarged heart accompanied by impaired contractile function and a risk of sudden cardiac death, and it is also one of the most common causes of heart failure in humans (Hershberger et al., 2013; McNally et al., 2013). The management of DCM necessitates a multifaceted approach, encompassing pharmacotherapy to enhance cardiac function and prolong survival, lifestyle modifications to maintain cardiac health, the implantation of devices such as pacemakers and implantable cardioverter defibrillators (ICDs) for arrhythmia management, and, in severe cases, heart transplantation (Kummeling et al., 2015; Halliday et al., 2017; Fatkin et al., 2019). While each of these therapeutic modalities offers distinct advantages in managing DCM, they are also associated with limitations and challenges, including medication side effects, surgical risks, and post-transplant immunosuppression issues. Consequently, ongoing research and therapeutic innovation are critical for improving treatment outcomes and quality of life for DCM patients. A notable genetic factor contributing to DCM is mutations in the RNA binding motif protein 20 (*RBM20*) gene. Individuals with mutations in this gene typically experience an earlier onset of DCM, a higher likelihood of progressing to end-stage heart failure, and an elevated mortality rate (Jordan et al., 2021; Kornienko et al., 2023). ABEs were employed to successfully correct *RBM20* gene mutations (RBM20^R634Q^, RBM20^R636S^) in iPSCs, achieving a high editing efficiency of up to 92% (Figure 6G) (Nishiyama et al., 2022). Building on this achievement, researchers developed a Rbm20^R636Q^ mutant mouse model. Subsequently, by intraperitoneally injecting AAV9 carrying ABEmax-VRQR-SpCas9, the researchers successfully restored cardiac function in the afflicted mice and prolonged their lifespan (Nishiyama et al., 2022). This offers a promising therapeutic approach for treating DCM and other diseases caused by mutant genes.

### 5.8 CD3δ severe combined immune deficiency

CD3δ severe combined immune deficiency (SCID) is a rare and severe hereditary disorder of the immune system, characterized by a profound impairment of immune system function (Picard et al., 2015). This disorder is related to developmental defects in the immune system and primarily involves genetic variations in the *CD3δ* gene, which is crucial for the assembly of the T-cell receptor (Garcillán et al., 2021). The absence of this gene precipitates a marked immunodeficiency. Bone marrow transplantation (or hematopoietic stem cell transplantation) serves as the principal therapeutic intervention for various forms of SCID, including CD3δ SCID (Grunebaum et al., 2006; Marcus et al., 2011; Fabio et al., 2020). This approach is distinguished by its capacity to afford enduring immune system reconstitution. Nevertheless, it is beset by several limitations, notably the challenge of identifying an appropriate donor, the elevated risks inherent in the transplantation procedure, and the possibility of graft rejection. In contrast, gene therapy represents a groundbreaking therapeutic avenue, fundamentally targeting the genetic aberrations responsible for disease by reinstating normal immune function. The application of ABE within HSPCs has been demonstrated to rectify the pathogenic mutations in the *CD3δ* gene associated with CD3δ SCID (Figure 6H) (McAuley et al., 2023). This correction reestablishes the T-cell developmental capabilities in HSPCs. Moreover, the sustained presence of amended hematopoietic stem cells post-transplantation presents a viable, one-off therapeutic avenue for individuals afflicted with CD3δ-SCID (McAuley et al., 2023). In the pathogenesis of SCID, dysfunctional *RAG1* and *IL2RG* genes play a critical role (Suzuki et al., 2012; Gan et al., 2021). Zheng et al. have developed a technique employing the CBE4max system to deactivate the *IL2RG* and *RAG1* genes, thereby successfully generating an immunodeficient monkey model (Zheng et al., 2023). These monkeys exhibit severely compromised immune systems, characterized by lymphocytopenia, atrophy of lymphoid organs, and an absence of mature T cells. The deployment of such immunodeficient monkeys can significantly augment the efficacy of preclinical trials, laying a robust groundwork for the advancement of biomedical science and its subsequent clinical translation.

### 5.9 Spinal muscular atrophy

Spinal muscular atrophy (SMA) is identified as a rare and severe hereditary neurological disorder, predominantly presenting in infancy or early childhood (Sugarman et al., 2012; Finkel et al., 2017). SMA results from homozygous deletion or mutation in the survival motor neuron 1 (*SMN1*) gene, leading to progressive muscle atrophy and weakness, ultimately causing impaired motor function (Prior, 2010; Finkel et al., 2017). *SMN2*, a human homologous gene to *SMN1*, produces a limited amount of functional SMN protein, but its stability is compromised by an exon deletion (Lorson et al., 1999). This variant protein partially compensates for SMN1 loss, alleviating spinal muscular atrophy symptoms to some extent. Recent years have witnessed significant advancements in the therapeutic landscape for SMA, with key treatments comprising antisense oligonucleotides such as nusinersen, AAV-mediated gene therapy onasemnogene abeparvovec, and the oral small molecule drug risdiplam (Yeo et al., 2024). Nusinersen modulates the splicing of the *SMN2* gene, thereby augmenting the production of full-length SMN protein, demonstrating potential in improving muscle functionality and enhancing survival prospects in patients (Finkel et al., 2017; Mercuri et al., 2018). Nonetheless, this therapeutic approach necessitates periodic intrathecal administration and the significant financial burden of the therapy. Onasemnogene abeparvovec, distinguished as the inaugural gene therapy sanctioned for SMA, facilitates persistent SMN protein expression via a singular intravenous administration, designed to curtail the advancement of the disease (Mendell et al., 2017; Mendell et al., 2021). However, its disadvantages encompass the substantial one-time treatment cost and the need for further observation to ascertain long-term effects and safety. Risdiplam, the inaugural oral therapy for SMA, acts as an SMN2 mRNA splicing modifier, facilitating the production of more full-length SMN protein (Baranello et al., 2021; Masson et al., 2022). Its benefits include ease of use and continuous drug delivery, yet it also faces challenges regarding high treatment costs and the evaluation of long-term efficacy and safety. As innovative therapeutic approaches for SMA are developed, the challenges associated with these treatments, particularly in terms of their invasive nature and the need for sustained intervention, become apparent. Against this backdrop, the application of base editing for the effective correction of aberrant splicing in the *SMN2* gene, thereby amplifying the expression of SMN protein in SMA patients, is emerging as a promising therapeutic strategy (Lin et al., 2020; Alves et al., 2023). An optimized D10-ABE base editing strategy (ABE8e-SpyMac and P8 sgRNA) targeting the mutation site in the *SMN2* gene attained an average T6>C conversion rate of 87% in Δ7SMA mice (Figure 6I) (Arbab et al., 2023). This approach enhanced motor function and prolonged the average lifespan in mice afflicted with the disease. Moreover, the synergistic application of this strategy with nusinersen has shown compatibility, augmenting the therapeutic outcomes (Arbab et al., 2023). This integrative approach holds significant potential for future clinical applications in treating spinal muscular atrophy.

## 6 Prospects

Within the burgeoning landscape of biomedical research, base editing has surfaced as an innovative breakthrough technology, particularly through the development of DNA and RNA base editors derived from the CRISPR system. The distinguishing feature of these technologies lies in their precision in modifying specific nucleotides on DNA or RNA, often without inducing double-strand breaks on DNA (Porto et al., 2020). This characteristic significantly diminishes the risks associated with insertions or deletions, commonly observed in conventional gene editing approaches. Base editing demonstrates immense potential in altering specific gene sequences and offers novel therapeutic possibilities at the post-transcriptional level. This transformative capability positions base editing as a promising strategy for treating a diverse array of genetic diseases, encompassing hematological disorders, neurodegenerative conditions, and some rare ailments. Nonetheless, to transition these technologies into clinical practice, extensive research and validation of their safety and efficacy are imperative. These considerations are crucial in driving the transition of base editing technology from laboratory settings to clinical applications and are of paramount importance for the future evolution of this field. Finally, we discuss several key considerations for base editing in the treatment of genetic diseases from different perspectives.

### 6.1 Off-target effects

Although base editors can achieve correction and modification of individual genes or segments, off-target effects remain an uncertain factor that they face. These effects are categorized into two types: expected off-targets, occurring in genomic regions with high sequence similarity to the target site, and unintended off-targets in unrelated areas (Zhang et al., 2015; Pacesa et al., 2022). Such effects could lead to genomic instability and disrupted gene functions, making the resolution of these off-target effects a crucial aspect of base editing. Off-target detection plays a crucial role in unraveling the off-target mechanisms of gene editing systems. Several off-target detection methods have been validated in assessing the specificity of the CRISPR/Cas9 system. These include sgRNA off-target prediction tools like Cas-OFFinder (Bae et al., 2014) and CFD (Doench et al., 2016), techniques such as GUIDE-seq (Tsai et al., 2015) for capturing DNA double-strand break sites, and *in vitro* strategies like Digenome-seq (Kim et al., 2016) and CIRCLE-seq (Tsai et al., 2017). In a comprehensive analysis using WGS, researchers evaluated the BE3, HF1-BE3, and ABE base editing systems in plants (Jin et al., 2019). They discovered that BE3 and HF1-BE3 induced a substantial number of SNVs in the rice genome, predominantly C-to-T mutations (Jin et al., 2019). Indeed, the study revealed that most of these additional SNVs did not coincide with off-target sites predicted by existing software, such as Cas-OFFinder, highlighting a disparity between the observed mutations and the predictive abilities of the software. The development of innovative off-target detection methods and the refinement of base editing tools are effective strategies that have propelled the advancement of gene editing technologies. A key breakthrough in this domain is the GOTI (genome-wide off-target analysis by two-cell embryo injection) technology, which is instrumental in detecting random off-target sites that were previously undetectable by conventional methods (Zuo et al., 2019). This enhancement in sensitivity significantly bolsters the precision of gene editing. In a related development, Lei et al. introduced Detect-seq, an unbiased off-target detection technique noted for its high sensitivity, specificity, and non-preferential nature (Lei et al., 2021). Detect-seq facilitates the detection of off-target sites induced by CBE across the whole genome, offering a more comprehensive understanding of gene editing impacts. Furthermore, the development of DeepABE and DeepCBE by Park and Kim represents a significant contribution (Park and Kim, 2023). These deep learning-based computational models are adept at accurately predicting the editing efficiency and outcomes of ABE and CBE, thereby streamlining the application of these tools in genome editing. In addition to these detection technologies, Xiong and colleagues have demonstrated remarkable efficacy with their SAFE (simple and fast off-target elimination) strategy for CBE/ABE (Xiong et al., 2023). SAFE enables efficient and low off-target base editing not only in plant models like rice and arabidopsis but also in human and yeast cells, underscoring its versatility. In conclusion, advancements in off-target detection and the enhancement of base editing tools are pivotal for propelling the field of gene editing forward. By intensifying research on off-target factors, mitigation strategies, and off-target detection technologies, we are optimistic that significant progress in overcoming off-target effects will be achieved in the near future. This focus not only promises to refine the precision of gene editing techniques but also enhances their safety and efficacy for clinical applications.

### 6.2 High efficiency and specificity

The advancement and practical application of base editing technology heavily depend on enhancing both the efficiency and specificity of base editing systems. High editing efficiency and specificity are essential because they ensure that the system can precisely and effectively alter the target sequence to bring about the intended genetic modifications. However, this efficiency is subject to influence by a myriad of factors, including the intrinsic activity of the enzyme, the modalities of its delivery, and the nature of the target site chosen for modification. To address these issues, researchers continually improve the design and performance of editing systems, including developing more specific enzyme variants, refining delivery methods, and optimizing the selection of modified target (Komor et al., 2017; Koblan et al., 2018; Huang et al., 2019; Gaudelli et al., 2020; Richter et al., 2020; Segel et al., 2021; Wang et al., 2023b; Kreitz et al., 2023). Additionally, the advent of high-throughput sequencing technologies and sophisticated machine learning models has led to the creation of online prediction tools (Zhang et al., 2023a; Kim et al., 2023). These tools serve as invaluable resources in guiding the selection of optimal base editing tools. They provide insights into the potential editing efficiency and off-target effects of various systems, thereby aiding in the optimization of their performance. Through innovative research, the development of advanced tools, and the utilization of predictive technologies, the goal is to tailor base editing systems that are both highly efficient and specific, paving the way for their safe and effective application in clinical settings.

### 6.3 Immunogenicity and safety

The potential of base editing tools to induce cytotoxic effects or trigger immune responses is a significant factor limiting their feasibility in clinical applications. Although the *in vitro* editing efficiency of current base editing systems has significantly improved, broader potential applications will require *in vivo* editing. The use of this technology *in vivo* comes with challenges, one of which is the immune response to Cas9. As an exogenous microbial-derived nuclease, Cas9 may elicit human adaptive immune responses, posing potential obstacles to the safety and effectiveness of base editors when used for therapeutic purposes in patients. This concern was highlighted by the work of Charlesworth and colleagues, who found antibodies against SaCas9 and SpCas9 in human serum donors (Charlesworth et al., 2019). This finding indicates the potential for both humoral and cell-mediated adaptive immune responses to Cas9 in humans, underscoring the need to consider the human immune system’s impact on CRISPR/Cas9-based therapies during clinical trials. Additionally, the interaction of Cas9 with cellular components can influence DNA repair mechanisms. Research by Xu and colleagues discovered that Cas9 interacts with the KU86 subunit of the DNA-dependent protein kinase complex, affecting the repair of DNA double-strand breaks through the non-homologous end joining pathway (Xu et al., 2020). Such interactions could have implications for the safety and efficacy of CRISPR/Cas9-based treatments. Enache and his team found that overexpression of the Cas9 protein can activate the p53 pathway in various cell lines, leading to the selective enrichment of p53-inactivating mutations (Enache et al., 2020). The study also indicates that the Cas9-induced activation of p53 in cell lines is likely to interfere with the results of genetic and chemical screens. Given these findings, it is clear that our understanding of the safety risks and mechanisms underlying CRISPR/Cas9 system usage is still evolving. Consequently, precise validation through laboratory studies and preclinical assessments is essential when advancing the clinical application of CRISPR/Cas9-derived base editing technologies. Employing such an integrative approach enables the maximization of the potential of base editing technologies within the healthcare domain, while concurrently safeguarding patient safety and wellbeing.

### 6.4 Clinical trials and ethical considerations

The maturation and standardization of base editing technologies have mitigated associated risks and ambiguities, thereby catalyzing their extensive deployment in clinical settings (Table 4). Clinical trials currently underway encompass a spectrum of genetic disorders, including but not limited to homozygous familial hypercholesterolemia, sickle cell disease, β-thalassemia, and T-cell acute lymphoblastic leukemia (Amit et al., 2022; Chiesa et al., 2023; Hughes-Castleberry, 2023; Naddaf, 2023). As base editing technology continues to evolve, its application in the clinical landscape is expected to expand, encompassing a wider range of genetic disorders. As the scope of base editing technology broadens, encompassing an increasing variety of genetic disorders, its potential to profoundly affect human health becomes more pronounced. This expansion highlights the need for stringent ethical scrutiny concerning the application of the technology. Key ethical considerations encompass the long-term effects of gene editing on individuals and society, demanding comprehensive risk evaluations and ethical deliberations before implementation (Coller, 2019; Rothschild, 2020). Furthermore, the equity and accessibility of gene editing technologies have come under scrutiny, raising concerns about the potential for creating disparities in health, social, and economic advantages among individuals, thereby influencing public acceptance (Coller, 2019). Moreover, the rapid development and clinical application of base editing technology challenge existing legal and ethical frameworks, requiring updates and enhancements to policies to protect patient rights while promoting scientific innovation. In summary, the development of base editing technology presents unprecedented opportunities for the treatment of genetic diseases, but the accompanying ethical considerations and challenges cannot be overlooked. Through ongoing scientific research, ethical scrutiny, and policy support, we hope to fully realize the immense potential of base editing technology in the treatment of genetic diseases, ensuring patient safety and rights.

**TABLE 4:** Clinical trials of base editing.

Disorder	Drug	Strategy	Delivery	Target genes	Status
Heterozygous familial	VERVE-101	Inhibition of gene expression	*In vivo* LNP	PCSK9	Phase Ib
Hypercholesterolaemia					
Heterozygous familial	VERVE-102	Inhibition of gene expression	*In vivo* GalNAc-LNP	PCSK9	Phase Ib
Hypercholesterolaemia					
Homozygous familial Hypercholesterolemia	VERVE-201	Inhibition of gene expression	*In vivo* LNP	ANGPTL3	Preclinical
T-cell acute lymphoblastic Leukemia	BE-CAR7	Multiple base editing eliminates gene expression	*Ex vivo* HSCs	TRBC, CD52, and CD7	Phase 1
Sickle cell disease/β-thalassemia	BEAM-101	Activation of fetal hemoglobin	*Ex vivo* T cells	HBG	Phase 1/2
Relapsed or refractory T-cell acute lymphoblastic leukaemia/T-cell lymphoblastic lymphoma	BEAM-201	Multiple base editing eliminates gene expression	*Ex vivo* T cells	CD7, TRAC, PDCD1 and CD52	Phase 1/2
Glycogen storage disease 1a	BEAM-301	Gene Correction	*In vivo* LNP	R83C	Preclinical
Alpha 1-Antitrypsin Deficiency	BEAM-302	Gene Correction	*In vivo* LNP	E342K	Preclinical
Transfusion-dependent	CS-101	Activation of fetal hemoglobin	*Ex vivo* HSCs	HBG	Trial (IIT)
β-thalassemia					

## 7 Conclusion

While base editing technology must be approached with caution in addressing various challenges, its immense potential in treating human genetic diseases is undeniable. A multitude of research findings have substantiated significant advancements achieved by base editing technology in treating specific genetic disorders. Future research directions should prioritize enhancing the precision of base editing, minimizing off-target effects, and developing safer, more efficacious delivery mechanisms. As we advance base editing technology, interdisciplinary collaboration becomes an indispensable component, involving the joint efforts of biologists, clinicians, ethicists, legal experts, and policymakers to ensure that technological advancements are achieved on the foundation of respecting human ethical principles. Through sustained research and innovation, we are confident that base editing technology will become a key force in transforming the treatment landscape for genetic diseases.

## Figures and Tables

**Table:** 

**aa**	Amino acids
**AAV**	Adeno-associated virus
**ABE**	Adenine base editor
**ABP**	Aptamer/aptamer-binding protein
**Ad**	Adenovirus
**ADAR**	Adenosine deaminases acting on RNA
**AP**	Apurinic/apyrimidinic
**AYBE**	A-to-Y transversions (where Y = C or T) base editors
**BE1**	rAPOBEC1–XTEN–dCas9
**BE2**	rAPOBEC1-XTEN-dCas9-UGI
**BE3**	rAPOBEC-XTEN-nCas9-UGI
**BE4**	rAPOBEC1-XTEN-nCas9-2UGI
**BH4**	Tetrahydrobiopterin
**CBE**	Cytosine base editor
**ceRBE**	Compact RNA base editor
**CGBE**	C-to-G base editor
**cGMP**	Cyclic guanosine monophosphate
**CHuMMMS**	Humanized aniridia mouse model
**CIS**	Contractile injection systems
**CRISPR/Cas9**	Clustered regularly interspaced short palindromic repeats/CRISPR-associated protein 9
**CRISPR-SKIP**	Achieves permanent exon skipping by introducing C to T or A to G mutations at splice acceptor sites in genomic DNA
**dCas9**	Dead Cas9
**DCM**	Dilated cardiomyopathy
**DMD**	Duchenne muscular mystrophy
**DMD** ^**E30mut**^ **, DMD** ^**Q1392X**^	Humanized duchenne muscular mystrophy mouse model
**Dmd** ^E4*^	A mouse model, which mimics the cardiac pathology of duchenne muscular mystrophy, exhibiting progressive cardiac dysfunction similar to that in human patients
**dsDNA**	Double-stranded DNA
**eA3A**	Engineered human APOBEC3A
**eBE**	Enhanced BE
**eCIS**	Extracellular contractile injection systems
**ERG**	Electroretinogram
**EMA**	European Medicines Agency
**eVLPs**	Engineered DNA-free virus-like particles
**FDA**	United States Food and Drug Administration
**FH**	Familial hypercholesterolemia
**GBE**	Glycosylase base editor (APOBEC-nCas9-UNG)
**gGBE**	A deaminase-free glycosylase-based guanine base editor
**GOTI**	Genome-wide off-target analysis by two-cell embryo injection
**HbF**	Fetal hemoglobin
**HDR**	Homology-directed repair
**HF-BE3**	High-fidelity base editor
**HF-Cas9**	High-fidelity SpCas9 variant
**HIV**	Human immunodeficiency virus
**HL**	Hearing loss
**HPFH**	Hereditary persistence of fetal hemoglobin
**HSC**	Hematopoietic stem cell
**HSPC**	Hematopoietic stem/progenitor cell
**ICD**	Implantable cardioverter defibrillator
**ICV**	Intracerebroventricular
**IDLV**	Integration-deficient lentiviral vector
**indel**	Insertions and deletion
**iPSC**	Induced pluripotent stem cell
**IRD**	Inherited retinal disease
**LCA**	Leber congenital amaurosis
**LDL**	Low-density lipoprotein
**LDL-C**	Low-density lipoprotein cholesterol
**LNP**	Lipid nanoparticle
**LV**	Lentivirus
**MEK or MAP2K**	Mitogen-activated protein kinase kinase
**mdx** ^**4cv**^	Duchenne muscular mystrophy mouse model
**MPG**	N-methylpurine DNA glycosylase
**mxABE**	Mini-dCas13X-mediated RNA adenine base editing
**nCas9**	Nickase Cas9
**NHEJ**	Nonhomologous end joining
**NLS**	Nuclear localization signal
**nt**	Nucleotide
**PAH**	Phenylalanine hydroxylase
**Pah** ^**enu2**^	Phenylketonuria mouse model
**PAL**	Phenylalanine ammonia-lyase
**PAM**	Protospacer adjacent motif
**PANCE and PACE**	Phage-assisted non-continuous and continuous evolution
**PAX6**	Paired box 6
**PCSK9**	Proprotein convertase subtilisin/kexin type 9
**PEG**	Polyethylene glycol
**PDEβ**	Phosphodiesterase
**PKU**	Phenylketonuria
**PRPF31**	Pre-mRNA processing factor 31
**PRPH2**	Peripherin 2
**PVC**	Photorhabdus Virulence Cassette
**Rbm20** ^**R636Q**^	Dilated cardiomyopathy mouse model
**RESCUE**	RNA editing for specific C to U exchange
**rd10**	A commonly used model for retinitis pigmentosa
**rd12**	Leber congenital amaurosis mouse model
**RHO**	Rhodopsin
**RNA-Seq**	RNA sequencing
**RNP**	Ribonucleoprotein
**RP**	Retinitis pigmentosa
**RPE**	Retinal pigment epithelium
**RPE65**	RPE-specific 65-kDa protein
**SAFE**	Simple and fast off-target elimination
**SCD**	Sickle cell disease
**SCID**	Severe combined immune deficiency
**SEND**	Selective endogenous encapsidation for cellular delivery
**sgRNA**	Single-guide RNA
**SMA**	Spinal muscular atrophy
**SMN1**	Survival motor neuron 1
**SNC**	Silicon nanocapsule
**SNV**	Single nucleotide variation
**SORT**	Selective organ targeting
**ssDNA**	Single-stranded DNA
**TALEN**	Transcription activator-like effector nuclease
**TAM**	Targeted AID-mediated mutagenesis
**TLS**	Translesion synthesis
**TMC1**	Transmembrane channel-like 1
**tsAAV**	Trans-splicing adeno-associated virus
**UDG**	Uracil DNA glycosylase
**UGI**	Uracil glycosylase inhibitor
**Ung**	Uracil-N-glycosylase
**VLP**	Virus-like particles
**WGS**	Whole-genome sequencing
**ZFN**	Zinc-finger nuclease
**Δ7SMA**	Spinal muscular atrophy mouse model
